# Predicting Adaptive Behavior in the Environment from Central Nervous System Dynamics

**DOI:** 10.1371/journal.pone.0003678

**Published:** 2008-11-07

**Authors:** Alex Proekt, Jane Wong, Yuriy Zhurov, Nataliya Kozlova, Klaudiusz R. Weiss, Vladimir Brezina

**Affiliations:** Fishberg Department of Neuroscience, Mount Sinai School of Medicine, New York, New York, United States of America; Indiana University, United States of America

## Abstract

To generate adaptive behavior, the nervous system is coupled to the environment. The coupling constrains the dynamical properties that the nervous system and the environment must have relative to each other if adaptive behavior is to be produced. In previous computational studies, such constraints have been used to evolve controllers or artificial agents to perform a behavioral task in a given environment. Often, however, we already know the controller, the real nervous system, and its dynamics. Here we propose that the constraints can also be used to solve the inverse problem—to predict from the dynamics of the nervous system the environment to which they are adapted, and so reconstruct the production of the adaptive behavior by the entire coupled system. We illustrate how this can be done in the feeding system of the sea slug *Aplysia*. At the core of this system is a central pattern generator (CPG) that, with dynamics on both fast and slow time scales, integrates incoming sensory stimuli to produce ingestive and egestive motor programs. We run models embodying these CPG dynamics—in effect, autonomous *Aplysia* agents—in various feeding environments and analyze the performance of the entire system in a realistic feeding task. We find that the dynamics of the system are tuned for optimal performance in a narrow range of environments that correspond well to those that *Aplysia* encounter in the wild. In these environments, the slow CPG dynamics implement efficient ingestion of edible seaweed strips with minimal sensory information about them. The fast dynamics then implement a switch to a different behavioral mode in which the system ignores the sensory information completely and follows an internal “goal,” emergent from the dynamics, to egest again a strip that proves to be inedible. Key predictions of this reconstruction are confirmed in real feeding animals.

## Introduction

Recordings from the central nervous system reveal a rich repertoire of dynamical activity, with a multitude of dynamical components on many time scales [1]–[4]. Following a reductionist strategy as well as practical necessity, these recordings of CNS dynamics are very often obtained from parts of the CNS, or even the whole CNS, *in vitro*. *In-vitro* analysis has certainly elucidated many of the cellular mechanisms that generate the dynamics. But to understand the functional significance of the dynamics, *in-vitro* analysis can be expected to be insufficient. The CNS with its dynamics has evolved to produce adaptive behavior, behavior that promotes the survival and reproduction of the animal, in the animal's environment. The CNS is thus functionally connected to the environment, both in its sensory and its motor capacity. At minimum, therefore, we need to consider how the CNS dynamics that we observe *in vitro* might correspond to the dynamics of sensory stimuli and behavioral acts in the environment.

**Figure 1:**
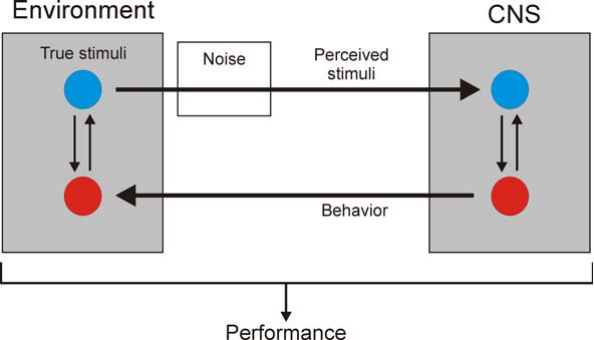
The coupled dynamical system of the CNS and environment, as discussed and modeled in this paper. The CNS (right) has intrinsic dynamics, schematized here by the blue and red circles and the arrows of interconversion between them that may represent, for example, the dynamics of the evolution toward ingestive and egestive steady states in the *Aplysia* feeding CPG that are described in the Results. These CNS dynamics then complement, in a sense explored in this paper, the structure and dynamics of the environment relevant to the production of adaptive behavior (left). The CNS and the environment are bidirectionally coupled. The CNS perceives the true stimuli present in the environment, but only through noisy sensory channels (left to right arrow). The CNS then generates the behavior in, and thereby modifies, the environment (right to left arrow). For simplicity, all noise within the system is lumped here, as in the modeling in this paper, into just one sensory noise source. The performance of the adaptive behavior emerges from the operation of the entire coupled system of both the CNS and the environment.

The dynamics of the CNS and environment are not so easily separable, however. In recent years, a dynamical-systems approach to neuroethology [5]–[8] has emphasized that the nervous system does not simply receive stimuli from the environment, or produce behavior in it, in a unilateral manner. Rather, the reciprocal sensory and motor interactions couple the CNS and the environment into a larger dynamical system (Figure 1). It is the dynamics of the entire coupled system that produce the adaptive performance, and that are selected for in evolution. Within the coupled system, the dynamics of a subsystem such as the CNS can be very different from those that the subsystem exhibits in isolation. Thus observations *in vitro*, while revealing what dynamics the CNS is intrinsically capable of, may not even show the dynamics that are actually instantiated in it in behavior, much less what their functional significance is. To learn this, we need to study the entire coupled system of the CNS and environment.

One way to do this, and one that ultimately will be required to test the understanding reached with any other approach, is to record *in vivo* from whole animals behaving in their natural environment. The complexity of the dynamics that can emerge when the CNS and environment are coupled together, however, suggests that any observations, whether *in vitro* or *in vivo*, will have to be embedded in a framework of computational modeling and analysis [6], [9].

The coupling suggests, at the same time, a computational strategy. For successful performance of a behavioral task by the coupled system, the dynamics of its CNS and environmental subsystems must, in some, perhaps complex manner, complement each other (Figure 1). The particular dynamics of any given CNS will not, indeed cannot, complement all environments. Rather, we may expect the CNS dynamics to be adapted to complement a particular subset of environments, those in which the animal, performing that behavior, has evolved. And, given the CNS dynamics, we should be able to predict which environments these are. We can embed a model of the intrinsic dynamics of the CNS, derived from the *in-vitro* observations, in a range of simulated environments and evaluate the performance of a suitable task. Success will identify the features of the environment to which the CNS dynamics are adapted, reveal the dynamics that are actually instantiated in the coupled system during the behavior, and allow us to examine the functional roles of various dynamical components. In this manner we should be able to reconstruct the entire system by working outward from the observations that we already have, of the CNS dynamics *in vitro*. This is the strategy that we pursue in this paper.

(In the real animal, of course, the coupling between the CNS and the environment is filtered through the body, whose dynamics will therefore have to be included in any completely realistic reconstruction. Here, in our first attempt at this problem, we will neglect these dynamics, but return to them in the Discussion.)

An analogous reconstruction of the entire CNS-environmental system from a given part of it is often done in the opposite direction. Given a task in a particular environment, the aim is to construct or indeed evolve a neural controller that will perform the task [10]–[15]. We, in contrast, start with the real controller that the animal uses and wish to predict from its properties the task and environment that it controls.

Here we carry out this computational reconstruction in the feeding system of the sea slug *Aplysia californica*. This classic, well-studied “simple” system [16], [17] allows the sensory-motor loop between the CNS and the environment to be closed in a relatively tractable fashion. Prominent dynamics on multiple time scales have recently been described in the feeding CNS *in vitro* (see Results). Here, by embedding a model of those dynamics in a simulated feeding environment, we examine their functional significance in the entire reconstructed system. We find that the combination of dynamical components in the system allows the behavior both to respond efficiently to environmental stimuli and, when necessary, to disregard them and follow an emergent, internal goal.

## Results

### *Aplysia* feeding behavior

*Aplysia* eat seaweed, often in the form of long fronds or strips [16], [18]. Once the seaweed has been located and contacted, consummatory feeding is a rhythmic, cyclical behavior, and many cycles are required to ingest, in incremental fashion, a long seaweed strip [19]–[21]. The cycle period is of the order of seconds or tens of seconds. (Movies of the behavior can be seen on our Web site at http://inka.mssm.edu/~seaslug/movies.html.) Each cycle of the behavior is triggered by local contact of the mouth of the animal with the seaweed [16], [22], [23]. Ingestion occurs when the radula, the central grasping organ of the buccal feeding apparatus, protracts from the mouth open, closes to grasp the seaweed, and retracts to pull the seaweed into the mouth [16], [20], [24], [25]. This phasing can be reversed, however, so that the radula protracts closed, grasping seaweed that has been ingested but judged inedible, to egest it again [20], [26]. Indeed, the feeding apparatus can produce feeding movements that span the entire range of ingestive-egestive character from strongly ingestive through “intermediate” to strongly egestive [20], [22], [27]–[29].

**Figure 2:**
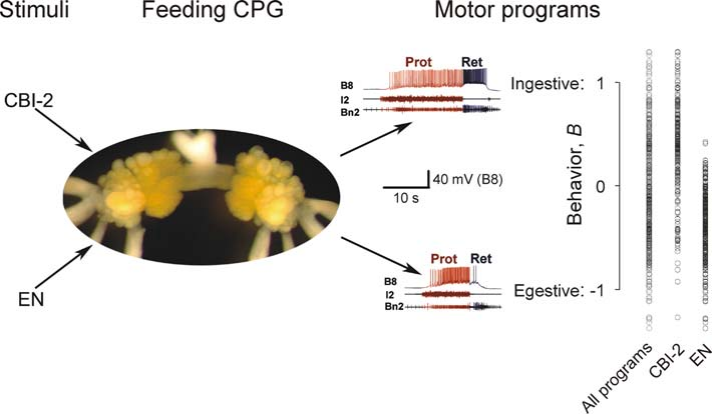
The *Aplysia* buccal feeding CPG *in vitro*. When driven by stimulation of the interneuron CBI-2 or of the esophageal nerve (EN), the CPG, residing in the paired buccal ganglia (photograph), generates feeding motor programs. The experimental records show two representative programs from the dataset in Figure 3, each with an intracellular recording from neuron B8 and extracellular recordings from two nerves, the I2 nerve and buccal nerve 2, whose activities are used as standard markers respectively of the protraction (red) and retraction (blue) phases of the program. It is then usual to classify the programs as ingestive or egestive depending on whether B8, a radula closer motor neuron, fires at higher frequency in the retraction phase (as in the top program) or the protraction phase (as in the bottom program), respectively. In this paper (as described in Text S1, Section 1.1), we have mapped the two B8 firing frequencies onto a single variable, the behavior *B*, that expresses the ingestive-egestive character of the programs along a single dimension, from *B* = 1 (most ingestive) to *B* = −1 (most egestive). On the right, all of the 466 programs in the dataset in Figure 3, then broken down into those elicited by CBI-2 or EN stimulation, are plotted along this dimension. Some programs exceed the limits of *B* = 1 or *B* = −1 because those limits are defined on average over all of the programs (see Text S1, Section 1.1).

The feeding movements are driven by patterns of neuronal activity, or motor programs, generated by a feeding central pattern generator (CPG) in the buccal ganglia [30]. The feeding CPG continues to generate these motor programs when the buccal ganglia are isolated *in vitro* (Figure 2). As *in vivo*, each program must be triggered by a stimulus. Two kinds of stimuli are used as analogs of the ingestive and egestive stimuli *in vivo*: electrical stimulation of the command-like interneuron CBI-2, which *in vivo* is activated when seaweed contacts the lips, and stimulation of the esophageal nerve (EN), which *in vivo* reports (among other things) the presence of inedible material in the esophagus [31]–[35]. The ingestive-egestive character of the programs is then quantified by comparing the frequencies of firing of the neurons B8, motor neurons that close the radula, in the retraction and protraction phases of the program [36]–[38]. If the B8 neurons fire, so that *in vivo* the radula would close, predominantly in retraction, the program is ingestive (for example, the top program shown in Figure 2), whereas if the B8 neurons fire predominantly in protraction, the program is egestive (the bottom program in Figure 2).

### Dynamics of the feeding CPG

It might be expected that the identity of the stimulus that triggers each motor program would at the same time specify the character of that program—that CBI-2 stimulation, the ingestive stimulus, would trigger ingestive programs, and EN stimulation, the egestive stimulus, would trigger egestive programs. *Aplysia* feeding would then be a purely stimulus-driven behavior. However, this is not the case. Figure 2, right, summarizes the ingestive-egestive character of 466 motor programs, elicited either by CBI-2 or by EN stimulation, recorded *in vitro* by Proekt et al. [39]—the dataset whose dynamics we will model and investigate in this paper. In anticipation of the modeling, we have already in Figure 2 mapped the B8 firing frequencies recorded by Proekt et al. onto a single normalized variable, the “behavior” *B*, that ranges from *B* = 1, indicating the most ingestive feeding motor program and, *in vivo*, the most ingestive feeding behavior, to *B* = −1, indicating the most egestive program and behavior (see supplementary Text S1, Section 1.1). Like the observed movements of the behavior *in vivo*, the motor programs span the entire ingestive-egestive range. Furthermore, both the CBI-2- and EN-elicited programs cover a large, and overlapping, part of the range. At different times, the same CBI-2 stimulation, in particular, can elicit a strongly ingestive or a strongly egestive program. Thus, as Proekt et al. [39], [40] concluded, the character of the motor programs is not directly specified by the stimulus. Neither is it random, however, or independent of these stimuli. Rather, it is specified by the internal state of the feeding CPG at the moment of stimulation, which evolves in response to the stimuli with well-defined, history-dependent dynamics.

**Figure 3:**
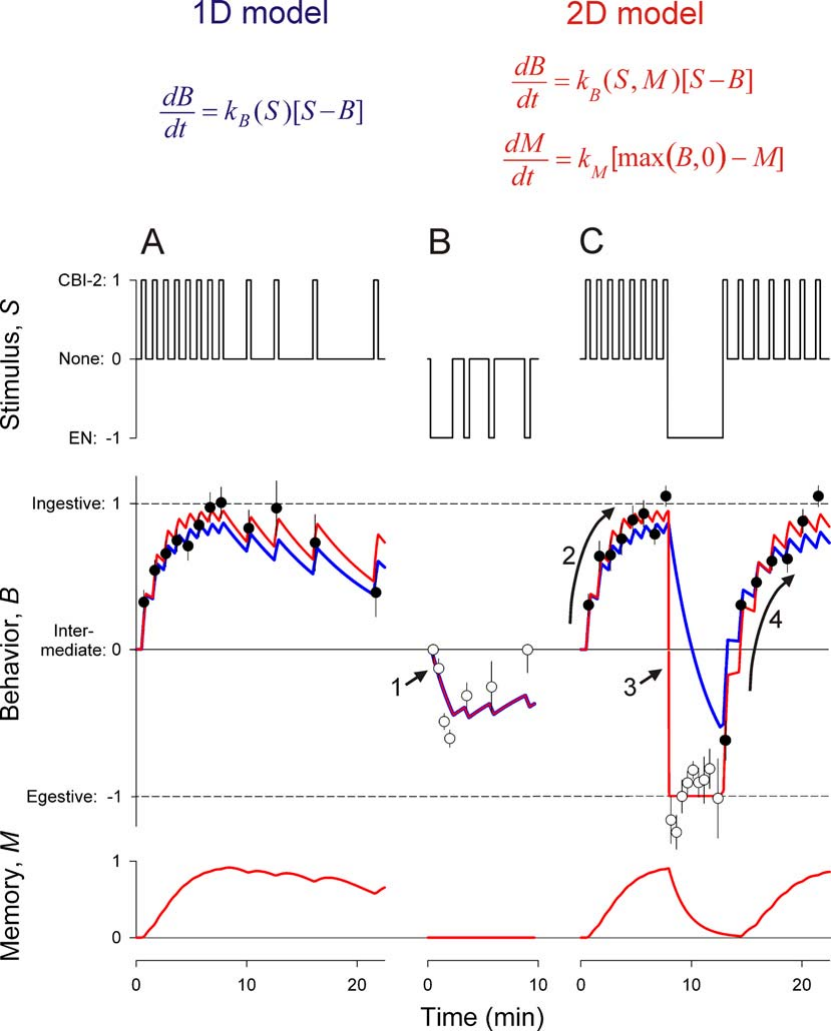
The dynamics of the *Aplysia* feeding CPG. Data from the experiments of Proekt et al. [39], already mapped onto our model variables. In the buccal feeding CPG preparation *in vitro*, Proekt et al. stimulated either CBI-2 alone (*A*) EN alone (*B*), or CBI-2 with an embedded period of EN stimulation (*C*) in the pattern shown here, represented by the stimulus variable *S*. From each motor program elicited by the stimulation, Proekt et al. measured the B8 firing frequencies in protraction and retraction (see Figure S1 and Text S1, Section 6.1), here mapped onto the single ingestive-egestive dimension of the variable *B*. Each filled or empty circle (CBI-2 or EN stimulation, respectively) represents the mean±SE of 6–18 programs; altogether the dataset contains 466 programs. The red curve is the best fit (see Text S1, Section 1.4) of the 2D model (equations summarized above; for complete model specification see Text S1, Section 1.3). The blue curve shows the corresponding behavior of the 1D model, not an independent fit to the data but rather simply the behavior of the 2D model with the memory *M* set to 0 (see Text S1, Section 2).

These dynamics are revealed when the motor programs in Figure 2 are plotted over time in Figure 3. Proekt et al. performed three types of experiments, stimulating CBI-2 alone (Figure 3*A*), EN alone (*B*), or CBI-2 with an embedded period of EN stimulation (*C*), in the pattern represented by the “stimulus” variable *S*. In each case, the first programs were intermediate, with the behavior *B* close to zero. Then, as the stimulation continued, the programs progressively evolved in the ingestive direction, toward *B* = 1, with CBI-2 stimulation (filled circles), or in the egestive direction, toward *B* = −1, with EN stimulation (empty circles). When the stimulation was discontinued, the programs relaxed back toward *B* = 0. The evolution occurred over several minutes, over a number of programs and, *in vivo*, cycles of the feeding behavior, with what we will therefore call “slow” dynamics. When the programs were made strongly egestive by EN stimulation and the stimulation was then switched to CBI-2, the first CBI-2-elicited program remained strongly egestive, and subsequent programs evolved in the ingestive direction with the same slow dynamics (Figure 3*C*, segment “4”). In other words, simply the starting point of the slow evolution of the CBI-2-elicited programs was displaced in the egestive direction (compare segments “2” and “4” of Figure 3*C*). Interestingly, however, the converse switch from CBI-2 to EN stimulation switched the programs from strongly ingestive to strongly egestive essentially immediately, with fast dynamics (Figure 3*C*, arrow “3”), much faster than was their slow evolution with EN stimulation alone (compare arrows “1” and “3” in Figure 3, *B* and *C*). Evolution in the egestive direction was thus greatly accelerated by a previous ingestive history.

We modeled these dynamics with a simple differential-equation model. The slow dynamics can be fully explained (blue curve in Figure 3) by a very simple “1D” model with just one dynamical variable, *B* itself, that relaxes slowly toward steady states at *B* = 1, −1, and 0 in response to CBI-2, EN, and no stimulation, represented by *S* = 1, −1, and 0, respectively (blue equation in Figure 3; for details see Text S1, Section 2). *B* itself thus *is* the internal state of the feeding CPG as it is expressed in the ingestive-egestive character of the feeding motor programs and behavior. The 1D model fails at just one point: it cannot explain the one component of fast dynamics in the data. This requires a “2D” model with an additional dynamical variable, which we call the “memory,” *M*. To explain the data, *M* builds up with its own slow dynamics when *B*>0 and then, upon EN stimulation, accelerates the relaxation of *B* toward *B* = −1 (red equations in Figure 3; see Text S1, Sections 1.2 and 1.3). *M* thus “remembers” ingestive behavior and modifies accordingly subsequent egestive behavior. The red curves of *B* and *M* in Figure 3 show the best fit of the full 2D model to the data.

These dynamics of the feeding CPG are not confined to the CPG itself, but emerge in the contractions of the various muscles and the phasing of the movements of the buccal feeding apparatus [29]. The question now is, what kind of behavior, in what kind of environment, are these dynamics adapted for?

### Task 1: prediction of an uncertain environment

One plausible role of such dynamics might be to predict the true state of the environment, so that the appropriate behavior can be produced. The true state of the environment is often uncertain. The “true” environmental stimuli may be incomplete and ambiguous, and they are perceived by the nervous system through limited and noisy sensory channels (Figure 1). Furthermore, the nervous system must often prepare now to execute the behavior later, in a future environment that is, by definition, unknown. Thus the behavior cannot simply be driven by the immediately perceived stimulus. Instead, the dynamics of the nervous system can act as an internal model that predicts what the true state of the environment will most likely be when the behavior is executed, and furthermore—when the dynamics are those of a complete sensory-motor system such as the *Aplysia* feeding CPG—it does so already in behavioral terms, by automatically producing the appropriate behavior. Proekt et al. [39] proposed that the slow dynamics of the *Aplysia* CPG act in this manner, integrating the perceived stimulus over time to estimate the true environment and consequently predicting conservatively that, when the next motor program is triggered, the true environment will most likely not have changed from that estimate and neither should the behavior. Thus, in Figure 3*C*, after the EN stimulation has made the programs egestive, the next program remains egestive even when it is triggered by CBI-2 stimulation.

**Figure 4:**
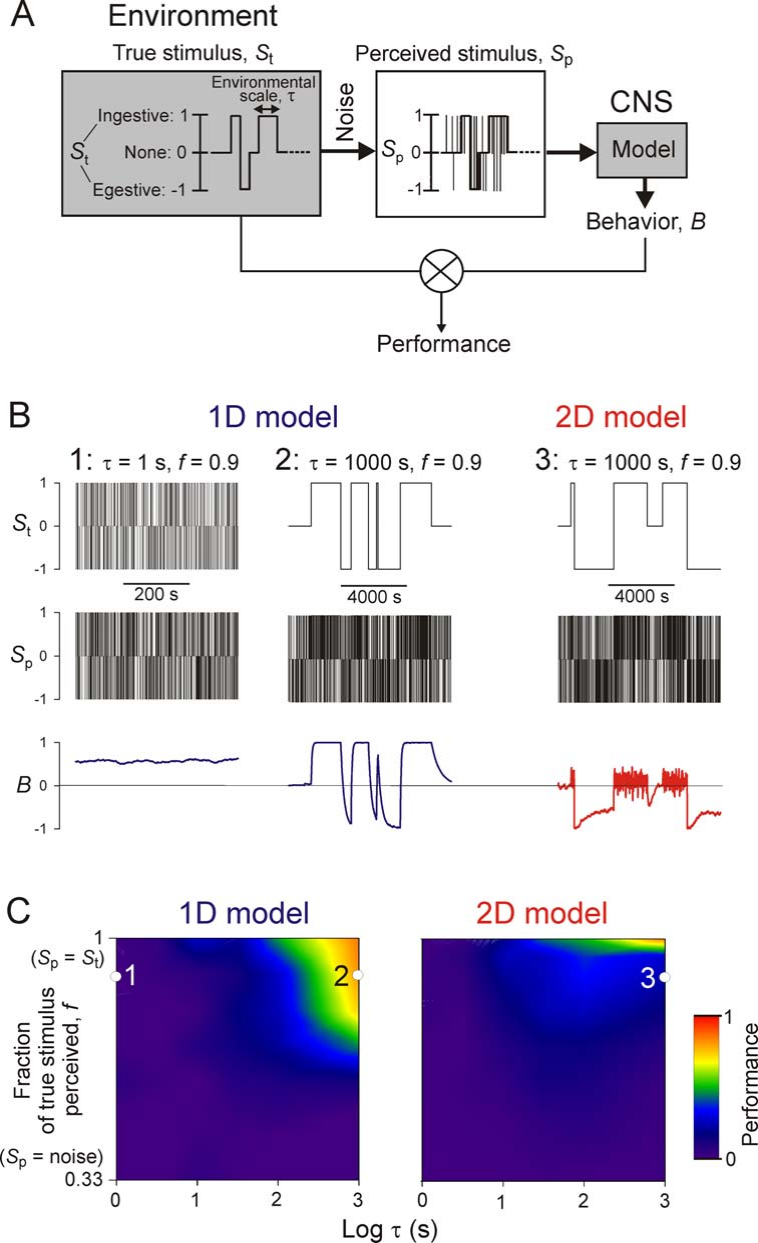
Simulations and performance of the 1D and 2D models in Task 1. *A*: Schema of the task, explained in Results. *B*: Steady-state excerpts from three representative simulations, with different values of the environmental parameters τ, the time scale of the environment, and *f*, the fraction of the true stimulus *S*_t_ that is apparent in the noisy perceived stimulus *S*_p_, and with either the 1D or the 2D model. In simulations 2 and 3, *S*_p_ is plotted sampled at 1/s, rather than 10/s as in simulation 1, to allow its structure to show through in these compressed plots. *C*: Performance of the 1D and 2D models, color-coded according to the scale shown on the right, over values of τ ranging from 1 to 1000 s (note the log scale) and *f* ranging from 1/3, where *S*_p_ is pure random noise with no information at all about *S*_t_, to 1, where there is no noise at all and *S*_p_ is identical to *S*_t_ (see Text S1, Section 3.2). The locations of the three simulations in *B* are marked.

How well do the dynamics of the *Aplysia* CPG in fact perform this role? We gave our CPG models such a predictive task in a simulated environment (Figure 4*A*). The environment consisted of a sequence of true stimuli, *S*_t_, randomly switching between ingestive, egestive, and none, represented by *S*_t_ = 1, −1, and 0, respectively, with durations drawn randomly from a Gaussian distribution with mean τ. To model the uncertain perception of the environment, the true stimulus *S*_t_ was then corrupted by fast random noise to give the perceived stimulus, *S*_p_, so that at any moment there was a given probability that if *S*_t_ was 1, say, *S*_p_ was 0 or −1. The CPG model was stimulated only with the perceived stimulus *S*_p_, yet its task was to match its behavior *B* as closely as possible to the true stimulus *S*_t_. Performance was defined simply as the average difference between *B* and *S*_t_ (for further details see Text S1, Sections 3.1 and 3.2).

Figure 4*B* shows three representative simulations and Figure 4*C* maps the performance of the two CPG models over a range of environments defined by the two parameters τ, the characteristic time scale of the environment expressed in the durations of the true stimuli *S*_t_, and *f*, the fraction of *S*_t_ perceived in *S*_p_—the degree of certainty of the environment. Cool colors represent poor performance, warm colors good performance. Consider first the 1D model, incorporating only the slow dynamics. When the environment was faster—that is, when *S*_t_ switched on average faster—than the slow dynamics of the model, *B* did not follow *S*_t_ at all (Figure 4*B*, simulation 1), resulting in poor performance (left side of Figure 4*C*, left). But when the environment was slower than the model dynamics, *B* tracked *S*_t_ well, ignoring a significant degree of obscuring noise (Figure 4*B*, simulation 2), resulting in good performance (top right corner of Figure 4*C*, left). Thus, indeed, by not responding to the perceived stimulus immediately but rather integrating it over time, the slow dynamics can extract from it a good prediction of the true environment, provided that the true environment, too, is slow. The slow dynamics are thus adapted to a slow environment.

The 2D model, however—the full model of the dynamics of the *Aplysia* feeding CPG—completely failed to perform this task (Figure 4*C*, right). With its fast dynamics in the egestive direction, the model tracked only egestive stimuli, not ingestive stimuli (Figure 4*B*, simulation 3). The model thus failed in a biologically significant manner: it failed to eat.

### Task 2: biologically realistic ingestion and egestion of seaweed strips

The failure of the 2D model in *all* environments defined by the two environmental parameters tested implied that, as far as these environments were concerned, Task 1 could not be the task to which the dynamics of the CPG are adapted. In developing a more relevant task, we were guided by a key feature of the dynamics themselves. While Task 1 was completely symmetric in the prevalence and order of ingestive and egestive stimuli, the observed CPG dynamics exhibit an asymmetric second-order coupling between ingestion and egestion. Egestion is facilitated by prior ingestion, but not vice versa. This presumably reflects the fact that, *in vivo*, egestion is evoked to expel inedible seaweed only if the seaweed has previously been ingested, but ingestion has no such prerequisite. We constructed a correspondingly asymmetric environment and task—indeed, by incorporating also the other basic facts of *Aplysia* feeding, a complete, biologically realistic feeding scenario.

**Figure 5:**
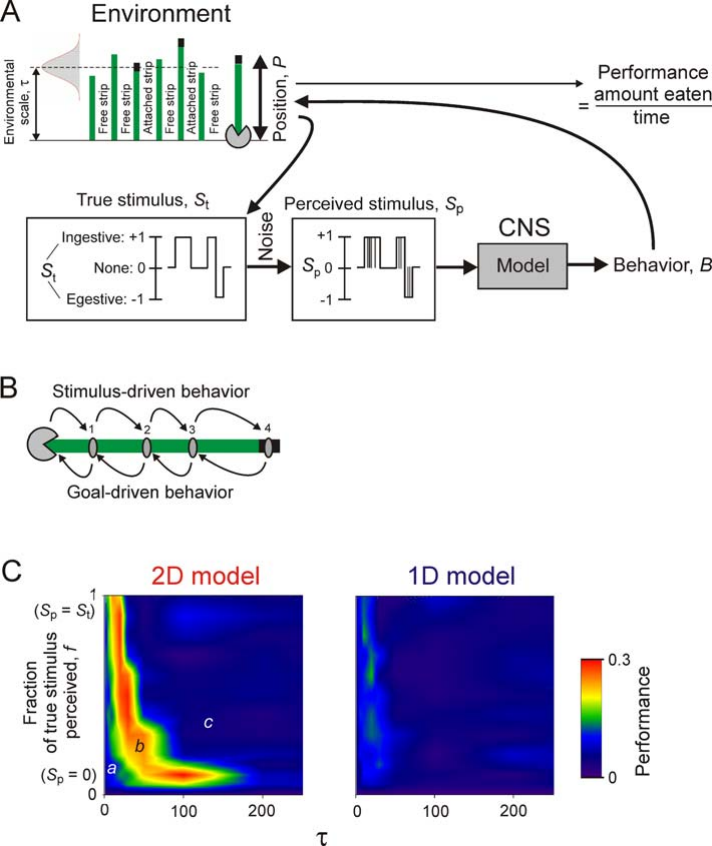
Performance of the 2D and 1D models in Task 2. *A*: Schema of the task, explained in Results. *B*: Local ingestive stimulus and global egestive goal oppose each other, driving the behavior in opposite directions along the same seaweed strip. *C*: Performance of the 2D and 1D models, color-coded according to the scale shown on the right, over values of τ ranging from 1 to 250 and *f* ranging from 0, where the true stimulus *S*_t_ is not perceived at all, to 1, where *S*_t_ is always fully perceived (see Text S1, Section 3.3).

In this scenario (Figure 5*A*; for details see Text S1, Section 3.3), the true environment consists of a large population of seaweed strips, with lengths drawn from a Gaussian distribution with mean τ, which the CPG model is to eat, necessarily sequentially, strip by strip. Intrinsically, all of the seaweed is edible, generating a true stimulus *S*_t_ = 1. However, because of the uncertain perception of the environment, as in Task 1, *S*_t_ reaches the model only intermittently, to a degree governed by the parameter *f*, as the perceived stimulus *S*_p_. Stimulated by *S*_p_, the model produces the behavior *B*, which now explicitly acts on the environment by translating to a rate of change of the position, *P*, on the current strip: ingestive behavior *B*>0 produces forward movement, and egestive behavior *B*<0 backward movement, along the strip. To eat, the model must move forward along the strip, and through the sequence of strips, as rapidly as possible since its performance is judged, in a biologically realistic manner, by the total length of seaweed eaten per time. In this scenario, therefore, the CPG model—now, indeed, essentially a simulated *agent* (cf. [6], [14], [41])—moves through the feeding environment, and consequently perceives that environment, in a manner that depends not only on the intrinsic properties of the environment, but also on its own actions in the environment.

This task would be straightforward, were it not for the fact that, while most of the strips are “free,” some of them (25% in the simulations in this paper) are “attached” at the end so firmly that, when ingested, they cannot be broken off. The attachment point (symbolized by the black color of the ends of the attached strips in Figure 5 and other figures) generates an egestive true stimulus *S*_t_ = −1 and the corresponding *S*_p_. The model must then—because this is tough seaweed that cannot be broken or cut anywhere along its length—egest the entire strip again, all the way back to the beginning, before it can continue to feed on another strip.

The model cannot simply avoid ingesting the attached strips in the first place, because at the beginning, and through the ingestion of their entire length until they are found to be attached, or not attached, at the end, all strips appear identical, all intrinsically edible, with *S*_t_ = 1. This is a consequence of the fact that the model, like real *Aplysia*, perceives only the purely local *S*_t_ and *S*_p_ just from the current point of contact with the environment. This fact has another interesting consequence. In egesting an attached strip, the model soon loses contact with the point of attachment where *S*_t_ = −1 and begins to move back over portions of the strip that, when the model moved over them earlier in the forward direction, generated, and now generate again, the intrinsic ingestive stimulus *S*_t_ = 1. Nevertheless, the model must continue to egest, following an egestive “goal” that is contrary not just to the perhaps misperceived stimulus *S*_p_, as in Task 1, but now even to the true stimulus *S*_t_. We will refer to this as “goal-driven” behavior, as opposed to the simple “stimulus-driven” behavior when the goal agrees with *S*_t_ (Figure 5*B*). Thus, in Task 2, it is no longer sufficient to predict the true state of the local environment at any moment, because the behavior appropriate to the local environment at any moment may not be the best behavior overall. Instead of a series of local predictions, the system must make, rather, a global prediction of the properties of the entire seaweed strip.

The 2D model, with both slow and fast dynamics, was able to perform Task 2, over a sharply defined range of environments, exceptionally well, indeed with performance approaching the theoretical maximum (see Text S1, Section 3.3) of ∼1/3 (Figure 5*C*, left). The 1D model, with only the slow dynamics, was not able to perform the task at all (Figure 5*C*, right).

### Performance emerges from an interaction of slow and fast dynamics

The region of high performance in Figure 5*C*, left, is conspicuously curved. This is because the performance depends not on τ or *f* separately, but on their product τ*f* (see supplementary Figure S3 and the accompanying Text S1, Section 6.3). The performance is low when either τ or *f* is small (in region “*a*”), and increases as τ*f* increases (from left to right, and bottom to top, through region “*b*”). The highest performance occurs around τ*f*≈17 (along the edge of region “*b*” facing region “*c*”). Above this (in region “*c*”), the performance abruptly collapses.

**Figure 6:**
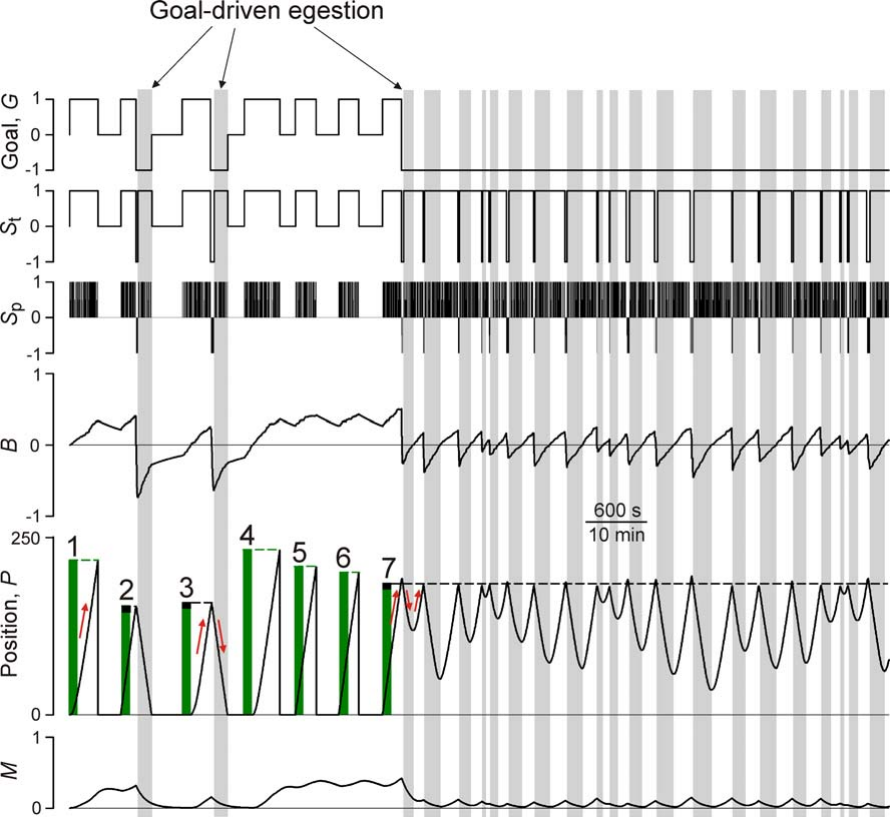
Characteristic modes of behavior of the 2D model in Task 2. Representative simulation at τ = 200, *f* = 0.1, showing (top to bottom) the goal *G*, the true stimulus *S*_t_, the perceived stimulus *S*_p_, the behavior *B*, the position *P* on the seaweed strip (the red arrows show the direction of movement), and the memory *M*. The vertical green bars show the lengths of seven seaweed strips presented at those times, some of the strips (1, 4, 5, and 6) being free and others (2, 3, and 7) attached, the latter indicated by the black color of their ends. The model proceeds through the simulation, completely ingesting all of the strips and, on finding strips 2 and 3 to be attached, egesting them again completely, but then fails to completely egest strip 7 and continues to oscillate back and forth on it, egesting part of it and then ingesting it again, essentially indefinitely. The vertical grey bars indicate periods of goal-driven egestion, that is, when, with *G* = −1, *B*<0 even though *S*_t_ = 1.

What behaviors of the model underlie this performance map, and how do the dynamics shape these behaviors? Figure 6 shows a simulation in which all of the characteristic modes of behavior of the model can be seen, by chance, at the same τ and *f*. (An interactive Java implementation of the simulation program, in which τ and *f* can be varied, can be found on our Web site at http://inka.mssm.edu/~nata/simulations/ode.html.)

Consider first the left half of Figure 6. The statistics of the environment specify a sequence of several free seaweed strips, three on average (e.g., strips 4, 5, and 6), between each pair of attached strips (strips 3 and 7). The slow dynamics of the model are slow enough to integrate the perceived stimulus *S*_p_ not just within each strip, but over the entire sequence of free strips. As a result, the behavior *B*, and so the rate of movement through the sequence, builds up in the ingestive direction to an amplitude that reflects the total amount of ingestive stimulus that has been perceived since the last attached strip—a function of the product τ*f* (see further Figure S3 and Text S1, Section 6.3). If τ*f* is small, for example if the strips are short (see Figure S2 and Text S1, Section 6.2), *B* remains small, the model moves slowly, and the performance is low. But with larger τ*f*, as in Figure 6, *B* can build up to a sufficiently large positive amplitude for the model to move forward at nearly the maximal rate, for near-maximal performance. Here, therefore, the slow dynamics are playing a role much like that in Task 1. Based on the accumulated perception of an ingestive environment, they set the state of the system for the most efficient continued ingestion.

At the same time, the memory *M* also builds up, enabling the fast dynamics that, when a strip eventually proves to be attached and generates an egestive stimulus, rapidly displace *B* to a sufficiently large negative value to begin rapid movement back along the strip.

Away from the point of attachment, however, the stimulus becomes ingestive and the slow dynamics begin to build *B* up in the ingestive direction again. Egestion, now goal-driven (vertical grey bars in Figure 6), nevertheless continues as long as *B* remains negative. If the entire strip is egested before *B* reaches zero, the model can go on to feed on further strips. Goal-driven egestion must therefore necessarily have been successful, repeatedly throughout the simulation, everywhere in the high-performance region “*b*” in Figure 5*C*, left. In Figure 6, strips 2 and 3 were thus successfully egested.

The egestion of strip 7, however, failed. In this case, conversely, *B* reached zero before the entire strip was egested. The right half of Figure 6 then shows the characteristic prolonged consequences of such a failure. As the model “forgets” the egestive goal and responds to the local ingestive stimulus again, the strip is ingested once more, and the model becomes trapped in oscillations back and forth over the same portion of the strip that can continue indefinitely. This failure is seen, naturally, with longer strips, more precisely above τ*f*≈17 (see Figure S3 and Text S1, Section 6.3), explaining the collapse of performance in region “*c*” of Figure 5*C*, left.

The slow relaxation of *B* from the initial negative value at the beginning of egestion back toward positive values can thus be pictured as acting like a count-down timer of the duration of the egestion, expiring when *B* reaches zero and thereby setting the maximal length of strip that can be egested. *M*, too, relaxes slowly during this period (Figure 6), and such timing would seem to be the natural function of a slow “memory.” But, in fact, the countdown is performed almost entirely by the intrinsic slow dynamics of *B* (Figure S4 and Text S1, Section 6.4). *M* contributes, instead, by controlling the initial negative value to which *B* is displaced by the fast dynamics—the value from which the countdown begins. That value depends on the speed of the fast displacement and *its* initial value. These, in turn, are products of the previous ingestion of the strip. The combination of fast and slow dynamics thus functionally links the successive phases of ingestion and egestion in a rather complex manner. Even though the model has only a few kinetic parameters, it would already be very difficult to predict its behavior, and to understand its adaptation to the environment, except by explicitly performing simulations and mapping its performance in a range of environments, as we did in Figure 5*C*, left.

In some environments, the model achieves near-maximal overall performance. This implies that not only the ingestion, but also the egestion is near-maximally efficient. In this regard, we can see that the superposition of the fast dynamics converts to efficient use what is normally an inefficient aspect of slow dynamics. Slow dynamics filter out spurious changes in stimulus, but by the same token they respond to real changes slowly, with a delay, transiently producing behavior that is inappropriate with respect to the true stimulus (see, e.g., Figure 4*B*). The fast dynamics exploit this property. They rapidly displace the state of the system so as to create a slow transient of behavior that, while inappropriate with respect to the true stimulus at that moment, is appropriate with respect to the overall goal.

The particular combination of fast and slow dynamics found in the *Aplysia* CPG appears to be tuned specifically to the range of environments in region “*b*” in Figure 5*C*, left. Given Task 2, these are the environments that this combination is adapted to. It is in this region, too, that the dynamics correctly predict the global properties of the entire seaweed strip, indeed, over the long run, act as if they instantiate a correct model of the statistics of the environment in which they are operating (Figure S5 and Text S1, Section 6.5).

### The CPG and the environment act as a coupled dynamical system

Although the intrinsic kinetic parameters of the CPG model are fixed, the dynamics that are actually observed are plastic because they are elicited from the model only through its reciprocal interaction with the environment. In response to stimuli from the environment, the model moves through the environment and thereby modifies, in turn, the stimuli that it receives. The dynamics, and their performance, are the product of the entire coupled system comprising both the CPG and the environment.

**Figure 7:**
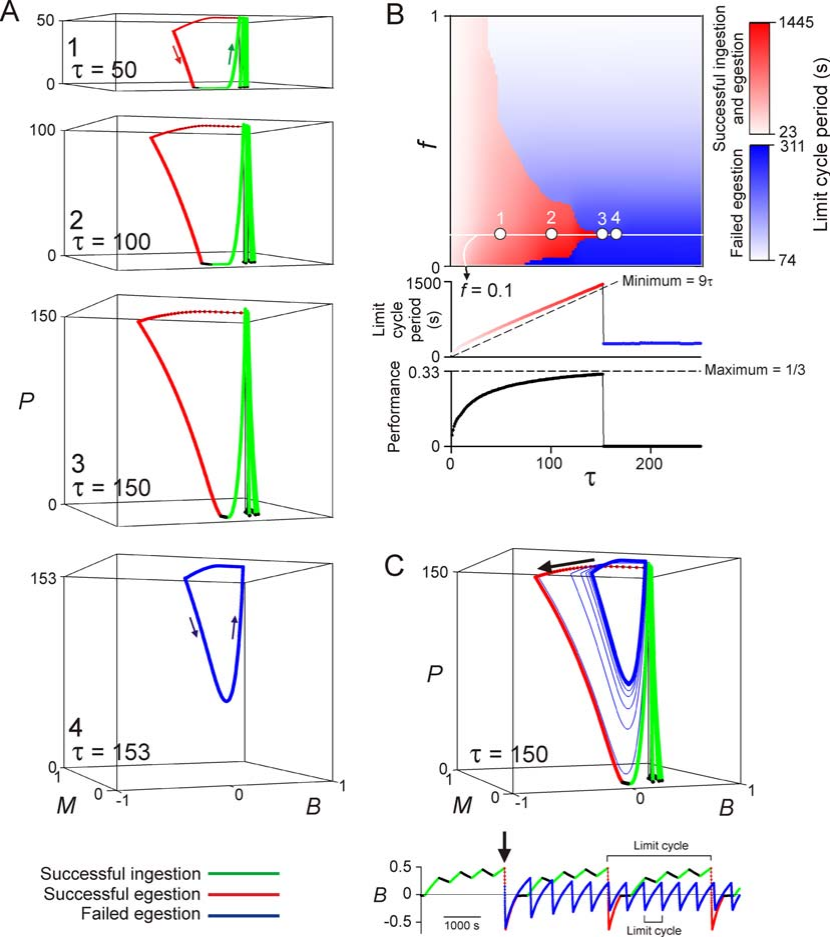
The CPG and the environment as a coupled dynamical system. All simulations in this figure used a 3-dimensional analytical system in which the fast noise in the perceived stimulus *S*_p_ was absorbed into the 2D model, which was then driven in Task 2 through the space spanned by the behavior *B*, the memory *M*, and the position *P* by the true stimulus *S*_t_ (see Text S1, Section 4). To reveal the canonical dynamics, all variability of the true environment was also eliminated in this figure, so that the environment consisted simply of repeats of the single canonical sequence of three free and one attached seaweed strips, all exactly of length τ. All simulations started from the initial conditions *B*(0) = 0, *M*(0) = 0, *P*(0) = 0. *A*: Representative steady-state limit cycle trajectories reached by the system with different values of τ, all with *f* = 0.1. The time required to complete one such cycle is the “limit cycle period” in this figure (see also *C*, bottom). *B*: Values of the limit cycle period, color-coded according to the scales shown on the right, over values of τ ranging from 1 to 250 and *f* from 0 to 1. In the red region, the limit cycle reached from the initial conditions *B*(0) = 0, *M*(0) = 0, *P*(0) = 0 consists of three successful ingestions and a successful egestion, as in simulations 1–3 of *A*; in the blue region, it consists of a failed egestion, as in simulation 4 of *A*. The locations of the four simulations in *A* are marked. Below is shown a representative section through the plot, again at *f* = 0.1, and under it the corresponding performance, given by 3τ/(limit cycle period) for a “successful” limit cycle and 0 for a “failed” limit cycle. With no variability, the smallest possible period of the successful limit cycle is exactly 9τ and the highest performance therefore exactly 1/3 (Text S1, Section 3.3). *C*: Over an intermediate range of τ, the two kinds of limit cycle coexist. Here, with τ = 150 and *f* = 0.1, a small perturbation moved the system from the successful to the failed limit cycle (see further Figure S6 and Text S1, Section 6.6). The corresponding time series of the behavior *B* is shown below.

Indeed, the performance map in Figure 5*C*, left, is the product of two fundamentally different dynamical modes in which the entire system operates: a “successful” mode at τ*f*<17 in which the model continues to progress through the environment (albeit, at small τ*f*, slowly so that the performance is low), and a “failed” mode at τ*f*>17 in which it ceases to progress. These two modes were the origin of the distinct behaviors seen in the left and right halves, respectively, of Figure 6. In Figure 7, the modes are demonstrated in an analytical version of the system in which all stochasticity is absorbed to reveal the fundamental dynamical structure (see Text S1, Section 4). Under these circumstances, as the simulation progresses, the system tends toward one of two limit-cycle attractors. Figure 7*A* shows examples of these attractors in the space of the three dynamical variables *B*, *M*, and *P*, the position on the seaweed strip. Figure 7*B* maps the cycle periods of the attractors over the range of environments, with a representative section through the map and the corresponding performance plotted underneath. At any particular *f*, short strips elicit the “successful” attractor, consisting of a series of successful ingestions and egestions (green and red, respectively, in Figure 7*A1–3*; red in 7*B*). But as the strips grow beyond a certain length, they suddenly elicit the “failed” attractor, the failed egestive oscillation back and forth on the same strip (blue in Figure 7*A4* and 7*B*). Thus, different environments induce the CPG model to express dramatically different dynamics—some, such as the failed oscillation, completely unanticipated from the kinetics of the CPG model alone—and consequently impart different dynamics to the environment itself, as seen from the vantage point of the model.

With strips of intermediate length, the two attractors coexist (Figure 7*C*; for details see Figure S6 and Text S1, Section 6.6). Switching between the two dynamical modes can then be induced by perturbations. Indeed, the rapid displacement of *B* by the fast dynamics at the beginning of egestion (black arrows in Figure 7*C*) can itself be thought of as such a perturbation. If *B* is displaced to a sufficiently negative value, relative to the length of the strip to be egested, then the system follows the successful attractor (green and red in Figure 7*C*). But if the displacement of *B* falls even slightly short, then the system spirals instead to the failed attractor (blue in Figure 7*C*). In the full system, such switching, from the successful to the failed mode and (less often) back, is then promoted by the stochasticity of the system, the variability of the lengths of the successive strips and the noise in the perceived stimulus that randomly perturbs the trajectories of the system around the underlying attractors (causing, for example, the variations in the successive oscillations in the right half of Figure 6, as compared with the unique attractor in Figure 7*A4*).

### Model predictions confirmed in real animals

In Task 2 we have described, in effect, the behavior and functional performance of a simulated *Aplysia* in a simulated feeding environment. Do we see similar behavior and performance when real *Aplysia* are feeding in a real environment?

First, how do the units of the model translate to real-world units? Although time is measured in seconds in the model, the units of length are formally arbitrary. They can, however, be converted to real units of length as follows. We know that the model ingests long seaweed strips at a maximal rate of 1 unit/s (Text S1, Section 3.3). In a real animal, the corresponding rate, although very variable, might be ∼0.5 cm per ingestive cycle lasting ∼5 s [21], or ∼0.1 cm/s. Thus 1 length unit is equivalent to ∼0.1 cm. What *f* might be in reality is unknown, but at *f* = 0.1, the maximal ∼170-unit length of a seaweed strip that can be ingested as well as egested successfully translates to ∼17 cm. This value is entirely consistent with the body size of adult *Aplysia californica* and the dimensions of the seaweed that they eat in the wild, both of which are of the order of centimeters to tens of centimeters [18], [42].

**Figure 8:**
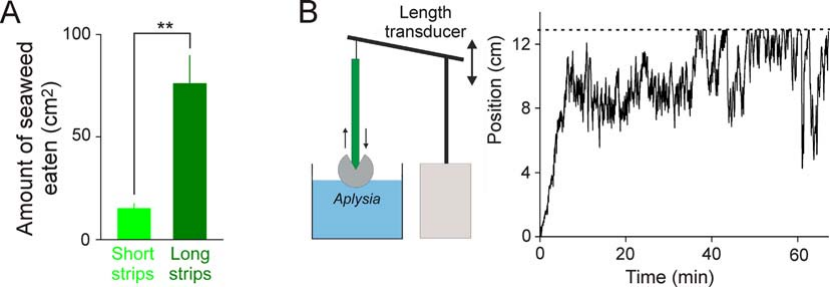
Two predictions of the model confirmed in real feeding *Aplysia*. *A*: Real *Aplysia* eat more free seaweed when it takes the form of long strips (corresponding to the high-performance region “*b*” in Figure 5*C*, left) than short strips (corresponding to the low-performance region “*a*”). The experiments were done as part of the work in [74], using the general methods described in [21], with animals chronically implanted with wire electrodes so that the cycling of the feeding CPG could be monitored. The experimental animal was placed in a ∼3-liter seawater tank with a surface area of 450 cm^2^, on which 150 cm^2^ of flat seaweed, cut into either 75 short (2×1 cm) or 10 long (15×1 cm) strips, was then randomly scattered. The animal was allowed to eat a complete “meal” [22] until the cycling of the feeding CPG spontaneously stopped; the number of remaining strips, and so the number of strips eaten, was then counted. From 21 such experiments with short strips and 12 experiments with long strips, the results (mean±SD) were as follows. Duration of the meal (min): short 53.5±19.4, long 49.0±15.1. Number of CPG cycles during the meal: short 144.7±71.2, long 198.1±98.0. Number of strips eaten: short 7.71±4.97, long 5.08±3.09. Total amount of seaweed eaten (cm^2^): short 15.42±9.94, long 76.25±46.33 (plotted in the figure). None of the differences between the short- and long-strip experiments were statistically significant (Mann-Whitney rank sum test) except the difference in the amount of seaweed eaten (whether over the entire meal, per cycle, or per minute), which was highly significant (*P*<0.01). *B*: Real *Aplysia*, like the model, can fail to egest a length of material completely and continue to oscillate back and forth on it indefinitely. Left: the experimental arrangement, described in detail in [21]. The animal ingested or egested material hanging vertically down to it from the arm of a length transducer, which recorded the movement. In this case, the material was 2 mm-diameter flexible plastic tubing, which (especially after prior arousal of the animal by seaweed chemical stimuli) appears to be perceived as an ingestive stimulus and is ingested [20], [25], and which, unlike seaweed, remains physically unaltered throughout long experiments such as this. Right: an example of the ingestive-egestive oscillations continuing for >1 hour. In the latter part of the sequence, the switch from ingestion to egestion was triggered by the apparent attachment of the material signaled by the increased tension when the transducer arm reached the limit of its range of motion (horizontal dashed line).

The performance map in Figure 5*C*, left, then predicts that long strips will be ingested more efficiently than short strips. Applying the conversion factor just determined, 15-cm strips, for example, should be ingested several-fold more efficiently than 2-cm strips. Indeed, when we allowed real animals to feed *ad libitum* on seaweed cut into either 2-cm or 15-cm strips, they ate, over a similar period of time, about 5-fold more seaweed in the latter case (Figure 8*A*).

The most striking prediction of the model is that, when a strip that has been ingested but now must be egested exceeds a certain length, the animal will “forget” the egestive goal and enter into the “failed” mode of ingestive-egestive oscillations back and forth on the strip. We tested this prediction with plastic tubing, a traditional seaweed substitute [20], [26] which *Aplysia* perceive as a (mild) ingestive stimulus and which remains physically unaltered, and so continues to present the same stimulus, upon repeated passage in and out of the animal. As in the model, the tubing was attached at the end so that it could be ingested, but it could not then be broken off and swallowed and had to be entirely egested again. We found that when the length of the tubing exceeded ∼10 cm, indeed, the ingestive-egestive oscillations appeared. Figure 8*B* shows a representative example in which the oscillations continued for more than an hour.

## Discussion

### The reconstruction strategy

In this work we have pursued a reconstruction strategy, which, from a known part of a system, seeks to deduce the other, unknown parts and so reconstruct the whole system. In this case we have sought to reconstruct the whole CNS-environmental system that produces an adaptive behavior from the known dynamics of the CNS. More commonly, the reconstruction of the CNS-environmental system is performed in the opposite direction: for a given behavioral task in the environment, the CNS controller is sought (for examples that are most comparable to our work, see [10]–[15]). Because degenerate solutions very often exist [9], [14], however, the reconstruction in that direction cannot guarantee that it will not yield a CNS controller that functions quite differently from the real biological one. This is one problem that we avoid, since we know that the CNS dynamics that we start from are in fact those of the real system. On the other hand, the reconstruction in our direction introduces the converse possibility of degenerate solutions in the environmental space, or, more generally, solutions that may depend also on other dimensions of the environment apart from those investigated. The selection of the environmental dimensions to investigate, like that of the task (see below), must therefore be guided to some extent by our basic understanding of the real system.

The reconstruction strategy relies on the constraints that the known part of the system imposes on the unknown parts, and will be more likely to yield meaningful results as these constraints increase. The constraints will increase as the parts of the system become more tightly intercoupled. With its closed-loop, feedback coupling between the CNS and the environment, our Task 2 apparently incorporates such constraints to a sufficient degree, judging by the emergence of the two distinct dynamical modes (Figure 7) and the sharply defined region of high performance in the environmental space (Figure 5*C*, left).

Since it proceeds by means of quantifying performance in a behavioral task, the reconstruction will be meaningful only if the task is meaningful. However, the task itself may not be completely understood. To some extent the task, too, may be inferred from the known part of the system that performs it. Here we thus inferred an ingestive-egestive asymmetry in the task from the ingestive-egestive asymmetry of the observed CNS dynamics. But usually a reconstruction of the whole system *and* the task will not be sufficiently constrained by the information available just within the system. External information will be required as further constraint. Here we used such external information by modeling Task 2 to agree with the basic facts of *Aplysia* feeding.

While simplified, Task 2 is realistic, except perhaps in one respect. When *Aplysia* encounter increased resistance when ingesting an otherwise edible strip of seaweed, they will attempt to cut or break off the strip at that point [19]. In Task 2 we have supposed that they do not succeed in doing so, and so must egest the entire strip again. This would be the case with tough seaweed or indeed plastic tubing (Figure 8*B*). In more general terms, Task 2 can be seen as a formalization of the regurgitation or vomiting scenario, in which the animal realizes only with a delay, after it has ingested a considerable amount of material, that that material is in fact indigestible or even harmful, and must egest all of the material again. Regurgitation or vomiting is observed in many kinds of animals [43] including at least some slugs [44], and its dynamics share many features with those that we have observed in Task 2 (see below).

### Roles of the slow and fast dynamics of the *Aplysia* feeding CPG

In the experimental work that we modeled here, the ingestive-egestive state of the *Aplysia* feeding CPG was found to have intrinsic dynamics that are generally slow, but exhibit one fast component when, after a period of ingestion, the feeding stimulus becomes egestive. In the realistic Task 2, we have observed and analyzed the functional consequences, and thus inferred the functional roles, of the slow and fast dynamical components and of their interaction.

The slow dynamics integrate the perceived feeding stimulus over long times, over multiple cycles of the feeding behavior and even from one ingested seaweed strip to the next. Conservatively, ignoring any fast changes in the perceived stimulus, the slow dynamics thus produce in each cycle behavior similar to that produced in the previous cycle. As Proekt et al. [39] proposed, this can be seen as the function of filtering the noisy perceived stimulus to extract an estimate of the “true” stimulus, the true state of the environment, and producing the behavior that is appropriate to it. Furthermore, since the function extrapolates from the past into the future, it can operationally be said to form a prediction or “expectation” of the future environment and an “intention” to produce the appropriate behavior [39]. With the slow dynamics, this function predicts a slowly changing environment, and so is adaptive if the environment is indeed slow. We demonstrated this function in isolation in our Task 1. Then in Task 2, as the animal ingests successive free seaweed strips, this function plays the key role of progressively building up the behavior in the ingestive direction for the most efficient continued ingestion.

In Task 2 the slow dynamics play also another, perhaps more surprising, role. When the true stimulus *does* change rapidly, the slow dynamics inevitably continue to produce for some time a transient of the old behavior, not yet reflecting the new stimulus. Normally this would represent an inefficiency inherent in the nature of slow dynamics. In Task 2, however, such a slow transient is in fact actively created and used to perform efficiently the most challenging part of the task—the phase of goal-driven egestion after the animal has found that an ingested seaweed strip is attached. Then, the slow transient continues to produce egestive behavior for a considerable time even after the local true stimulus generated by the animal's contact with the intrinsically edible seaweed has, once away from the point of attachment, resumed its ingestive character. In this egestive behavior, the system in effect expresses an estimate, no longer of the local stimulus, but of the global properties—the attached nature and the length—of the entire seaweed strip.

The transient is created by the fast dynamics. Having been enabled by the previous ingestion, the fast dynamics, on encounter with the point of attachment, rapidly displace the behavior to a strongly egestive state, the starting point of the slow transient.

Together, the slow and fast dynamics thus implement, in effect, a simple count-down timer. The fast dynamics set the initial value from which the countdown begins, and the slow dynamics govern the rate of the countdown. Both together determine how long the countdown—the phase of goal-driven egestion—lasts. This can be seen as a primitive analog of the function of interval timing that has long been studied in the vertebrate CNS [45], which, according to one proposal, may likewise keep track of the passage of time on the time scales of seconds and minutes through the decay of a slow memory [46].

Formally, the dynamics and their roles do not depend on the particular neurophysiological mechanisms that implement the dynamics within the CPG. Nevertheless, it is worth mentioning that recent studies have begun to reveal some of these mechanisms. Briefly, in the CPG some neurons appear to lie functionally on the input side and others on the output side, in that their activities track the ingestive-egestive character of the stimulus and of the behavior, respectively [40], [47]. The slow dynamics probably emerge in the connections between these two types of neurons through such mechanisms as activity-dependent synaptic plasticity [39], [40]. To some extent, also, alternative sets of neurons may become active as the character of the stimulus and/or behavior changes [38], [47], [48]. Finally, because these various neurons release in an activity-dependent manner different combinations of neuropeptides that modulate the activity of the CPG, the ingestive-egestive state of the CPG may in fact reside partly in its state of neuromodulation [38], [49].

### Stimulus- and goal-driven behavior

The different dynamical mechanisms that underlie ingestion and egestion, together with the asymmetry in the statistics of the environment where most seaweed strips are free and only a few are attached, mean that the phases of ingestive and egestive behavior are fundamentally asymmetrical.

Ingestion is stimulus-driven. The ingestive goal coincides with the ingestive true stimulus generated by the seaweed, and so matching the behavior to the true stimulus is all that is required. Because the slow dynamics integrate the stimulus from one seaweed strip to the next, as the animal ingests successive free strips it does so more and more efficiently, and becomes more and more primed to immediately begin ingesting the next strip that it encounters. The default state of the system is thus ingestive. The ingestion can go on indefinitely, as long as the ingestive stimuli continue to arrive.

Eventually an ingested strip is found to be attached, however. Then the dynamical timer mechanism executes a self-delimited, discrete phase of egestion that disregards, indeed opposes, the true ingestive stimulus and follows instead an internal egestive goal that emerges from the dynamics. This egestion has many of the characteristics of a stereotyped reflex or fixed-action pattern. Once triggered, it proceeds automatically. Its duration is preset by the amplitude of the initial egestive displacement by the fast dynamics, which, since they are enabled by the previous ingestive history, can to some extent tailor the duration to that history—if a great deal of seaweed has recently been ingested, the egestion will last longer. But otherwise the duration is relatively resistant to modification. If the egestion of the strip is completed early, the system nevertheless persists for the remainder of the duration in an egestive state in which (exhibiting a loss of “appetite”) it will not ingest another strip (this can be seen in Figure 6, and especially clearly following strips 18 and 21 in Figure S2). If, on the other hand, the duration is too short to egest the strip completely, the duration is not prolonged, but rather the entire egestive reflex is repeated anew (right half of Figure 6). Many of these characteristics are shared by vertebrate vomiting [50], which too is considered to be a complex reflex or fixed-action pattern that, indeed, may be orchestrated by a CPG-like network [43], [51].

Such interweaving of phases driven by an external stimulus and an internal goal is observed in the behavior of many other real animals, as well as their models [8], [11], [13], [14].

The ingestive and egestive phases impart opposite tendencies to the overall feeding performance mapped in the environmental space. The ingestion increases in efficiency as the scale of the environment, the average length of the seaweed strips, grows longer. The egestion, on the other hand, loses all efficiency when the strips become too long to egest completely. We confirmed both of these tendencies with real animals (Figure 8, *A* and *B*, respectively). Between the two tendencies, there is a relatively narrow range of environments (region “*b*” in Figure 5*C*, left) in which the overall performance is high, indeed near the theoretical maximum for Task 2. It is in this range that the CPG dynamics correctly estimate the global properties of the seaweed strips, indeed act as if they instantiate a correct model of the statistics of the environment in which they are operating (see Figure S5 and Text S1, Section 6.5). The seaweed lengths in this range are entirely consistent with those that *Aplysia* encounter in the wild. These are the environments that, according to our analysis, the dynamics of the *Aplysia* feeding CPG are adapted to, presumably because they have evolved in them. This conclusion reflects the basic concept that natural selection operates only on those sensory capabilities and behavioral acts that the animal actually expresses in its ecological niche, and tends to optimize those particular capabilities and acts even if others that normally are not expressed are thereby degraded (see, e.g., [8], Chapter 1).

### Dynamical modes of the entire coupled system

As has been emphasized in previous dynamical-systems work in neuroethology [5]–[8], it is not the dynamics of the CPG alone, but rather of the entire reciprocally coupled system of both the CPG and the environment, that produce the performance. Indeed, in Task 2, once the *Aplysia* “agent” is placed in the environment, the motions of the coupled system proceed completely automatically. Setting aside noise and variability, the entire system tends to one of two dynamical attractors (Figure 7). If the agent is placed in an environment of seaweed strips that are not too long, the system tends to a “successful” attractor in which the agent continues to ingest, and if necessary egest, one strip after another. If, however, the agent is placed in an environment of long strips, the system tends to a “failed” attractor in which the agent, remarkably, becomes trapped in an oscillation back and forth on the same strip. Guided by the modeling, we were in fact able to observe this failed mode of behavior in real animals (Figure 8*B*). The successful and failed modes of behavior represent emergent, collective properties of the entire dynamical system (see, e.g., [8], Chapter 6). They would not easily have been predicted by studying its parts, either the CPG or the environment alone, without our combined analysis.

### Further work

Three extensions of the present work naturally suggest themselves.

First, we have neglected in this work the dynamics of the body through which the interactions between the CNS and the environment necessarily pass [7]. The intrinsic dynamics of the musculature that performs the behavior, in particular, can considerably modify the dynamics of the behavior [52], [53]. In the *Aplysia* feeding system, the buccal musculature exhibits complex intrinsic dynamics, with slow and fast components of its own, that are due to the actions of neuromodulators released within the musculature as the behavior proceeds [54]–[57]. We were able to neglect these peripheral dynamics here because, to a first approximation, the dynamics of the CPG do still emerge in the contractions of the muscles and the phasing of the feeding movements [29]. In other words, the dynamics of the body appear to be relatively transparent to such basic features of the CNS activity as its ingestive-egestive character. Nevertheless, the peripheral dynamics must now be added to our model for a complete examination of the dynamics of the entire coupled system of “brain, body, and environment” [7]. Indeed, certain details of our modeling (see, e.g., Text S1, Section 3.3) can already be interpreted as a rudimentary differentiation between the ingestive-egestive character of the motor programs and that of the actual behavior of the animal.

Second, we have neglected, too, yet another component of the CPG dynamics: a large quasi-random variability of essentially all parameters of the motor programs, including their ingestive-egestive character, from one cycle to the next [58]. The CPG dynamics of Proekt et al. [39] that we modeled here represent just the average through this variability. This variability, too, emerges in the muscle contractions, feeding movements, and indeed the experimentally measured cycle-to-cycle performance of the feeding behavior [21], [59]. The variability must likewise now be added to the model. In preliminary simulations in which we added a simple model of such variability (Figure S7 and Text S1, Section 6.7), we found that the variability significantly broadened the high-performance region in the environmental space, especially (in the manner of the perturbations discussed in the Results) by allowing long attached strips, which otherwise would have triggered an indefinite period of the failed oscillations, to be successfully egested sooner or later. This is consistent with the idea that, in an uncertain feeding environment, the variability serves to implement a trial-and-error diversification of the feeding movements until a movement succeeds [21], [58]. From the perspective of variability, indeed, the plot of the performance in the environmental space can also be regarded as a plot of the variability in the feeding task that the CNS dynamics can deal with successfully.

Third, it should now be possible to connect the behavior of the model with that of the real *Aplysia* in more specific, mechanistic terms. The model makes specific predictions that can be tested. For example, in the experiments in Figure 8*A* the real *Aplysia*, just like the model, performed better with the long as compared to the short seaweed strips in terms of the overall measure of the amount of seaweed eaten over a long time. But in the model, this is specifically because, as the behavior is integrated by the slow dynamics progressively more in the ingestive direction, each cycle is more strongly ingestive. In the real *Aplysia*, we should therefore find, if we use a method like that in Figure 8*B* to continuously record the movement of the seaweed, that the better performance with the long strips is accounted for by a greater length of strip pulled in per cycle, rather than more cycles each pulling in the same length of strip.

Conversely, finer details of the real behavior can be added to the model. For example, the plastic tubing used in Figure 8*B*, although traditionally used for such experiments [20], [26], was clearly not physiological. These experiments must now be repeated with real seaweed, which the animal will presumably be able to cut, rather than need to egest completely, more often. The probability of cutting can be incorporated into the model. [Indeed, the model already contains a probability that the strip will break at any point, although in the simulations presented here that probability was set at a negligibly low value (see Text S1, Section 3.3).]

### Cognitive-like operations in simple circuits

Altogether, the *Aplysia* feeding system interprets sensory information in the light of past experience and the current functional goal of the animal to formulate predictions, “expectations,” and “intentions” concerning the immediate future [39] and express them in the optimal adaptive behavior. If performed by a mammalian CNS, these would be regarded as intelligent, “cognitive” functions. Yet here they are performed by a simple motor network, when coupled to the environment.

We can see the operational similarity, and use such terms as “expectations” and “intentions,” when both cognitive processes and processes that normally would not be considered cognitive are expressed in a common language, in particular that of dynamics [8], [41], [60]–[64]. In terms of dynamics, the system is no longer viewed as having any such specific internal representation as, for instance, of the fact that an attached seaweed strip of a certain length has been ingested and must now be egested. Rather, the dynamics of the system simply cause it to act so as to egest the strip. The dynamics have evolved to perform adaptive behavior, “the essence of intelligence” [5]. When operationally compared in this manner, many other simple systems can be seen to perform basic intelligent or cognitive-like operations [11], [13], [15], [65], [66]. Quite sophisticated operations of the same kind are performed even by bacteria and genetic, biochemical, and protein-protein interaction networks within cells [67]–[69]. In humans and animals that normally are credited with cognitive capabilities, by the same token, it may be that the cognitive operations, and other such high-level operations as the internal representations of the body and the environment in sensory-motor transformations [70]–[73], are in fact implemented in a distributed manner throughout the entire dynamical system: the nervous system, including its low-level circuits, the nonneural structures that are involved in the behavior, and the environment, whose structure may indeed serve as a “scaffolding” essential to many human cognitive capabilities [8], [60].

## Methods

All modeling and analysis of the modeling results was done in *Mathematica* (Wolfram Research, Champaign, IL) or during the preparation of the figures in SigmaPlot (SPSS, Chicago, IL). Specific details of the numerical integration methods are given in Text S1, Section 5. The experimental methods used in Figure 8 were closely based on those used previously by Lum et al. [21]; specific details are given in the legend to Figure 8.

## Supporting Information

Text S1 1. Construction of 2D model. 2. 1D model. 3. Modeling of environment, tasks, and performance. 4. Analysis of 2D model in Task 2. 5. Numerical integration methods. 6. Supplementary figure legends and discussion. 7. Supplementary references.
(0.24 MB PDF)

Figure S1 Experimental data and fit of the 2D model.
(1.24 MB TIF)

Figure S2 Low performance of the 2D model in Task 2 in a short environment.
(1.08 MB TIF)

Figure S3 Complete analysis of the shape of the region of high performance of the 2D model in the Task 2 environment.
(0.33 MB TIF)

Figure S4 The slow dynamics of the behavior *B*, rather than the decay of the memory *M*, determine the longest seaweed strip that can be egested.
(0.92 MB TIF)

Figure S5 Prediction of the environment by the 2D model in Task 2.
(1.71 MB TIF)

Figure S6 More detailed dynamical analysis of the success or failure of the ingestion and egestion of seaweed strips of different lengths.
(1.39 MB TIF)

Figure S7 Added variability enhances the performance of the 2D model in Task 2 simulations.
(1.64 MB TIF)
