# Supplementary material for: Predicting Adaptive Behavior in the Environment from Central Nervous System Dynamics
Source: PLoS One. 2008 Nov 7;3(11):e3678. doi: 10.1371/journal.pone.0003678 (PMC2576442; doi:10.1371/journal.pone.0003678)
Supplement: Text S1 — 1. Construction of 2D model. 2. 1D model. 3. Modeling of environment, tasks, and performance. 4. Analysis of 2D model in Task 2. 5. Numerical integration methods. 6. Supplementary figure legends and discussion. 7. Supplementary references. (0.24 MB PDF) [file pone.0003678.s001.pdf]

Proekt et al.

Predicting adaptive behavior in the environment from central nervous system dynamics

Supplementary Text

1. Construction of 2D model 2

1.1. Experimental data 2

1.2. Considerations for model formulation 3

1.3. 2D model 4

1.4. Fitting the model to the data 4

2. 1D model 6

3. Modeling of environment, tasks, and performance 7

3.1. General schema 7

3.2. Task 1 7

3.3. Task 2 8

4. Analysis of 2D model in Task 2 12

5. Numerical integration methods 14

6. Supplementary figure legends and discussion 15

6.1. Figure S1. Experimental data and fit of the 2D model 15

6.2. Figure S2. Low performance of the 2D model in Task 2 in a short environment 15

6.3. Figure S3. Complete analysis of the shape of the region of high performance of the 2D model in the Task 2 environment 16

6.4. Figure S4. The slow dynamics of the behavior B*, rather than the decay of the memory* M*, determine the longest seaweed strip that can be egested 17*

6.5. Figure S5. Prediction of the environment by the 2D model in Task 2 18

6.6. Figure S6. More detailed dynamical analysis of the success or failure of the ingestion and egestion of seaweed strips of different lengths 19

6.7. Figure S7. Added variability enhances the performance of the 2D model in Task 2 simulations 22

7. Supplementary references 23

1. Construction of 2D model

1.1. Experimental data

The data used to construct the model were those contained in Figures 1 and 2 of Proekt et al. [S1] and additional data from the same experiments. In the buccal feeding CPG preparation *in vitro* (Figure 2 of the main text), Proekt et al. stimulated either the interneuron CBI-2 or the esophageal nerve (EN) and characterized the ingestive-egestive nature of the resulting feeding motor programs. Following standard practice (see, e.g., [S2-S9]), Proekt et al. defined the protraction phase of each program to be coincident with a characteristic pattern of high-frequency spike activity recorded extracellularly in the I2 nerve, and the retraction phase with the subsequent pattern of high-frequency activity recorded in buccal nerve 2 (Bn2; see experimental records in Figure 2). As a readout of the ingestive-egestive nature of the program, Proekt et al. recorded intracellularly the firing of one of the motor neurons B8 and computed its mean firing frequency over the duration of the protraction phase and of the retraction phase. Since the neurons B8 are radula closer motor neurons [S3, S10], firing of B8 predominantly in retraction is used as the standard *in-vitro* indication of an ingestive program in which, in the intact animal, the radula would be closed in retraction to pull material (food) into the mouth, whereas firing of B8 predominantly in protraction is an indication of an egestive program in which the radula would be closed in protraction to push material out of the mouth ([S2, S3, S5-S9]; see records in Figure 2).

Figure S1, *A-C*, shows the entire dataset that was modeled. The upward grey bars show the frequency of B8 firing in retraction, and the downward bars in protraction, when CBI-2 and EN were stimulated as indicated by the pattern of the small white and black blocks, respectively, across the middle of the plot, in three different paradigms, either CBI-2 alone (*A*), EN alone (*B*), or CBI-2 with an embedded period of EN stimulation (*C*). Details of how the raw data were used to construct Figure S1 are given in Section 6.1.

The standard practice [S1, S9, S11] would be to represent these data in the 2-dimensional plane spanned by the B8 firing frequencies in protraction and retraction (Figure S1*D*). However, as examination of Figure S1, *A-C*, makes clear, the average trajectory of the system through the 2-dimensional plane was really only 1-dimensional. Either the protraction frequency varied while the retraction frequency remained at some minimum value (in Figure S1*B* and the middle of *C*), or the retraction frequency varied while the protraction frequency remained at a minimum value (in *A* and the beginning and end of *C*), but the two frequencies never varied simultaneously. This enabled us to map the entire dataset onto a 1-dimensional, normalized line (the "space of the model," L-shaped within the 2-dimensional plane as shown in Figure S1*D*) described by a single variable, B ("behavior"). $B = 0$ corresponds to the minimum frequencies in both protraction and retraction and represents an "intermediate" motor program character [S1]. From there, B can move in the positive direction, as the retraction frequency increases, toward $B = 1$, corresponding to some maximum value of the retraction frequency and the most ingestive program character, or B can move in the negative direction, as the protraction frequency increases, toward $B = -1$, corresponding to the maximum value of the protraction frequency and the most egestive program character (Figure S1*D*).

To actually scale the frequency values plotted in Figure S1, *A-C*, to the normalized values of B, we needed to know the minimum and maximum protraction and retraction frequency values at $B = 0$, 1, and -1. Taking the mean of all of the protraction frequency values in Figure

S1*A* and the beginning of *C*, which did not vary with the CBI-2 stimulation, we found the minimum protraction frequency to be 2.96 Hz (as indicated by the lower dashed line "$B = 0$"). Similarly, taking the mean of all of the retraction frequency values in Figure S1*B* and the middle of *C*, which did not vary with the EN stimulation, we found the minimum retraction frequency to be 0.97 Hz (upper dashed line "$B = 0$"). The maximum protraction and retraction frequencies, 6.62 Hz (dashed line "$B = -1$") and 8.39 Hz (dashed line "$B = 1$") respectively, were found in the course of fitting of the model to the data (see below). Using these four values, we converted the dynamically relevant parts of the data in Figure S1, *A-C*—that is, at each time point, the mean ± SE frequency represented by the bar either in protraction or in retraction, whichever was varying in response to the stimulation—into the normalized dataset seen in Figure 3, *A-C*.

1.2. Considerations for model formulation

We made the structure of the model no more complex than was necessary to reproduce the main features of the data. Formulation of the model was guided in particular by the following considerations:

(1) The model is based on the standard concept of dynamical variables whose motions are implicitly described by ordinary differential equations.

(2) Although the real motor programs are discretely articulated in time, with discrete protraction and retraction phases and discrete interprogram intervals, the modeled time t flows smoothly and the modeled variables are all time-continuous. In particular, $B(t)$ exists at every timepoint and can be interpreted as the ingestive-egestive character that a motor program would have if it were produced at that particular time. The stimulus input to the model, $S(t)$, similarly exists at every timepoint (see Figure 3).

(3) B ranges continuously between −1 and 1, as described in the previous section. S can only assume the discrete values of 1, −1, or 0, representing respectively CBI-2 stimulation, EN stimulation, and no stimulation in the data of Proekt et al., and more generally ingestive, egestive, and no stimulus input to the model.

(4) A model with only the one dynamical variable $B(t)$, driven by the input $S(t)$, is sufficient to reproduce much of the data, in particular its slow dynamical trends (see Section 2 below, the blue curve in Figure 3, and the main text). However, it fails at one critical point. When EN stimulation is applied to the system at $B = 0$, B decreases toward −1 slowly (arrow 1 in Figure S1*B*), but it does so much more rapidly (arrow 2 in Figure S1*C*) if there has been a previous history of CBI-2 stimulation and/or positive B. Disambiguation of these different motions of B from the same initial value requires a second dynamical variable, $M(t)$ ("memory").

(5) The memory M operates also on the increase of B toward 1 with CBI-2 stimulation. This is suggested by an observation (data not shown, but the dashed red trajectory in Figure S1*C* demonstrates this behavior in the model) that the previous history of CBI-2 stimulation and/or positive B accelerates not only the decrease of B toward −1 with EN stimulation, but also a subsequent return of B toward 1 if the stimulation is switched back to CBI-2 relatively soon. Again, from the same initial value of $B = 0$, B increases more rapidly (arrow 4 in Figure S1*C*) than it did with the same CBI-2 stimulation alone at the beginning of Figure S1*C* (arrow 3) or in Figure S1*A*. These different motions of B from the same initial value again require disambiguation, most simply, by the operation of M.

(6) However, if the stimulation is switched back to CBI-2 only after a longer period of EN stimulation, such as after 5 min in Figure S1*C*, the increase of B toward 1 is no longer any

more rapid (arrow 5) than it was when the CBI-2 stimulation was not preceded by any particular history (arrow 3). This suggests that the memory M is, in the simplest interpretation, only of CBI-2 stimulation and/or positive B, and not of EN stimulation and/or negative B, and furthermore that the memory has decayed after 5 min.

(7) There is little in the available data to indicate whether M is a memory of the CBI-2 stimulation or of the positive B. A model with either formulation would reproduce the data in Figure S1 and the two models would most likely behave and perform quite similarly throughout the simulations in this paper. We chose to make M a memory of the positive B as slightly more intuitive.

1.3. 2D model

The consideration in the previous section resulted in a model given by the equations

$$\frac{\mathrm{d}B(t)}{\mathrm{d}t} = k_B\left[S(t) - B(t)\right], \tag{S1}$$

$$\frac{\mathrm{d}M(t)}{\mathrm{d}t} = k_M\left[\max\left(B(t), 0\right) - M(t)\right], \tag{S2}$$

with $B(t) \in [-1, 1]$, $M(t) \in [0, 1]$, and $S(t) \in \{-1, 0, 1\}$, where

$$k_B = \begin{cases} k_x + k_{w1}M(t) & \text{if } S(t) = 1 \\ k_y & \text{if } S(t) = 0 \\ k_z + k_{w2}M(t) & \text{if } S(t) = -1 \end{cases},$$

and k_x = 0.02, k_y = 0.002, k_z = 0.00496, k_{w1} = 0.0368, k_{w2} = 2.93, and k_M = 0.01, determined as described in the following section.

1.4. Fitting the model to the data

First, since the value of the memory rate constant k_M was not well constrained by the available data, we set it *a priori* to 0.01, a value sufficiently high to satisfy consideration (6) of Section 1.2 above, but sufficiently low to satisfy, to some degree at least, consideration (5). In any case, the exact value of k_M did not appear to be critical for the behavior and performance of the model (Figure S4*A1*).

We then needed to determine the values of the remaining rate constants, k_x, k_y, k_z, k_{w1}, and k_{w2}. To do this, we ran the model by numerically integrating Equations S1 and S2 (see Section 5) with the stimulation pattern of the experiments in Figure S1 as the input $S(t)$, starting, since before each experiment the preparation had been for a prolonged period unstimulated and quiescent, from the initial conditions $B(0)$ = 0, $M(0)$ = 0. We repeated this with different candidate sets of the five rate constants plus two additional parameters (see below), evaluating the goodness of fit in each case by computing the squared error between the modeled $B(t)$ and the dynamically relevant data values (not just the bar means, but the underlying individual values) in Figure S1. To search through the 7-dimensional space of the values of the seven parameters, we first varied each of the values randomly or on a coarse grid over a wide range to identify regions of superior fit, then performed a systematic search on a fine grid or a gradient descent search through each such region. We first did this with any subset of the dataset in Figure S1 that allowed us to fit just a subset of the parameters (in Figure S1*B*, for example, just

k_y and k_z plus one of the additional parameters), but then finalized the best fit of all seven parameters simultaneously over the entire dataset in Figure S1.

The two additional parameters that it was necessary to fit together with the five rate constants were the maximum frequencies of B8 firing in protraction and retraction that mapped to $B = -1$ and $B = 1$, respectively, and so allowed the entire dataset in Figure S1 to be mapped with the appropriate scaling onto the space of B, as already described in Section 1.1. The best-fit values for these two parameters were 6.62 and 8.39 Hz, respectively.

The best fit of the model that we found is shown by the solid red curves in Figure S1 (and then reproduced in Figure 3). This fit, of the model with the rate constant values given in the previous section, has the least squared error, and so the least root mean square (RMS) error, between the modeled $B(t)$ and the dynamically relevant data values. The dataset in Figure S1 contains 466 dynamically relevant data values, with an overall mean of 5.96 Hz, in 43 bars. The RMS error of the fit with respect to the data values is 2.14 Hz, or 35.9%. However, the RMS error of the data values with respect to the means of their respective bars is almost as large, 1.89 Hz. Thus much of the error of the fit is irreducibly inherent in the scatter of the data: even if the modeled $B(t)$ passed exactly through the mean of each bar, it would still have an RMS error of 1.89 Hz, or 31.7%, with respect to the individual data values.

2. 1D model

The 1D model is given by the equation

$$\frac{\mathrm{d}B(t)}{\mathrm{d}t} = k_B\big[S(t) - B(t)\big], \qquad \text{(S3)}$$

with $B(t) \in [-1, 1]$ and $S(t) \in \{-1, 0, 1\}$, where

$$k_B = \begin{cases} k_x & \text{if } S(t) = 1 \\ k_y & \text{if } S(t) = 0 \\ k_z & \text{if } S(t) = -1 \end{cases} .$$

Thus, the 1D model is structurally identical to the 2D model but without the memory *M*. Since the rate constants k_x, k_y, and k_z are not modified by *M*, their intrinsic values must be somewhat different from those in the 2D model for the 1D model, too, to fit the data in Figure S1. However, for simplicity, and to explicitly reveal the contribution of *M* to the behavior and performance of the 2D model, the 1D model is shown throughout this paper with the same rate constant values, $k_x = 0.02$, $k_y = 0.002$, and $k_z = 0.00496$, as the 2D model. It is therefore precisely the 2D model but with $M(t) = 0$. In most parts of the dataset in Figure S1, the fit of this model is only slightly inferior to that of the full 2D model (Figure 3), or presumably that of the 1D model with the values of k_x, k_y, and k_z adjusted for best fit.

3. Modeling of environment, tasks, and performance

3.1. General schema

Construction of the environment, simulation of the behavior of either model within it, and evaluation of the model's performance involved a series of time-varying quantities, constructed ahead of the simulation or computed dynamically during it. In Task 1, each of these quantities derived from the previous one in a simple cascade but in Task 2 they had additional feedforward and feedback interactions.

(1) The "true" environment, governed by a single parameter, the "environmental scale" τ, was first defined.

(2) In Task 2, the true environment implied a time-varying "goal," $G(t)$, with respect to which the performance was evaluated in (7).

(3) In Task 1, the true environment itself constituted the "true stimulus," $S_t(t)$. In the more complex Task 2, the true environment combined with feedback from the position of the model in the environment, computed in (6), to generate $S_t(t)$.

(4) $S_t(t)$ was corrupted by noise to give the "perceived stimulus" $S_p(t)$. For the sake of computational and analytical simplicity, we chose to introduce the noise into the temporal rather than the amplitude dimension of the stimulus, preserving its three discrete levels of 1, −1, and 0 but corrupting the temporal occupancy of these levels. The degree of the corruption, more usefully expressed conversely as the degree to which $S_t(t)$ remained apparent in $S_p(t)$, was governed by the parameter f, the "fraction of the true environment perceived."

(5) With $S_p(t)$ as the input, the model produced the behavior $B(t)$.

(6) In Task 2, $B(t)$ was converted into a "position" $P(t)$ relative to the true environment.

(7) Finally, from two or more of the above quantities—$G(t)$, $S_t(t)$, $B(t)$, and $P(t)$, depending on the task—the performance $\mathbf{P}(t)$ was computed, a time-continuous quantity that was then more usefully summarized, however, in a single overall performance value at the end of the simulation.

The details of each of these steps were as follows.

3.2. Task 1

In Task 1, the characteristic of the environment that was of interest was temporal—essentially, the speed of the environment—and the environmental scale τ was a time scale with units of seconds.

Each simulation was performed with a particular fixed value of τ and a particular fixed value of f. Across simulations, τ ranged from 1 to 1,000 s, and f (for the reasons given below) from 1/3 to 1.

The waveform of $S_t(t)$ for the entire simulation was constructed by joining piecewise a sequence of intervals whose successive durations were drawn randomly from the positive part of a Gaussian distribution with mean τ and standard deviation τ, and whose successive amplitudes were chosen randomly with 1/3 probability to be 1, 0, or −1 (see Figure 4, *A* and *B*).

The waveform of $S_p(t)$ was then constructed by systematically modifying the amplitude of $S_t(t)$ on a much shorter time scale. The entire waveform of $S_t(t)$ was segmented into very short intervals whose successive durations were drawn randomly from an exponential distribution with a mean of 0.1 s. For each of these intervals, a random number, r, was drawn from the uniform

distribution on [0, 1]. If $r \le f$, the amplitude of $S_p(t)$ during the interval was set to the amplitude of $S_t(t)$ at the beginning of the interval, otherwise it was set randomly [i.e., depending on whether $f < r \le f + (1-f)/2$ or $r > f + (1-f)/2$] to one of the other two possible values (see Figure 4, *A* and *B*). This procedure implied that the meaningful range of f was from 1/3, where $S_p(t)$ was pure random noise with no information at all about $S_t(t)$, to 1, where there was no noise at all and $S_p(t)$ was identical to $S_t(t)$. Since the smallest τ used in the simulations in this paper was 1 s, the noise, on a time scale of 0.1 s, was always at least 10-fold faster than the true time scale of the environment.

With the waveform of $S_p(t)$ as the input $S(t)$, numerical integration of Equations S1 and S2 of the 2D model, or Equation S3 of the 1D model, starting from the initial conditions $B(0) = 0$, $M(0) = 0$, then produced the corresponding waveform of $B(t)$ (see Figure 4*B*).

The performance was computed as $\mathbf{P}(t) = B(t)S_t(t)$, then averaged over the entire duration of the simulation, except for the first 1000 s to exclude the transient. This formula computes, for $S_t(t) = 1$ or -1 only, how closely $B(t)$ reproduces that $S_t(t)$. **P** produced by the formula ranges from −1, signifying the largest possible mismatch between $B(t)$ and $S_t(t)$ [when $S_t(t) = 1$, $B(t) = -1$, and vice versa], to 1, signifying a perfect match [$B(t) = S_t(t)$]. **P** = 0 would be obtained if $B(t)$ were produced without any regard to $S_t(t)$ (randomly or indeed at any constant value if $S_t(t)$ is random). In practice, most Task 1 simulations had **P** > 0, and only the positive half of the range of **P** is plotted in Figure 4*C*.

With the stochastic $S_p(t)$, each simulation, even with the same τ and f, gave a somewhat different value of **P**. To reduce this variability, each simulation was run for 30,000 s, or 100 times longer than τ, whichever was longer. This was long enough to give a reasonably settled average value of **P** at the end of the simulation. In the plots in Figure 4*C*, furthermore, the results of 8 independent simulations have been averaged together at each combination of τ and f.

3.3. Task 2

In Task 2, the environment was defined in the first instance in spatial, rather than temporal, terms; the environmental scale τ was a length scale, with arbitrary units, although *a posteriori* roughly convertible to centimeters (see main text). The spatial dimension then unfolded into the temporal dimension as the model negotiated the spatial environment in time (see schema in Figure 5*A*).

As in Task 1, each simulation was performed with a particular τ and f. Across simulations, τ ranged from 1 to 1,000 (the most interesting phenomena occurred below 250 or 300, however, where many of the plots in this paper therefore end) and f from 0 to 1.

Similarly to Task 1, the true environment was constructed as a sequence of lengths drawn randomly from a Gaussian distribution with mean τ and standard deviation, in this case, τ/5. (In the early stages of this work, we experimented with different values of this and the other fixed parameters in this section, eventually settling on the final values given here.) The stimulus amplitudes assigned to the successive lengths were, however, then generated by additional second-order rules: (1) In strict alternation, successive lengths were assigned amplitudes of 1 and 0. In the biological interpretation (see main text), the former were “seaweed strips” and the latter intervals of no stimulus between the strips. The first length in each simulation was always a seaweed strip. (2) At random, 1/4 of the seaweed strips in the entire sequence were designated as “attached,” while the other strips remained “free.” Formally, this was represented by inserting

after each attached strip a length equal to its length but with an amplitude of −1.

This sequence of the true environment defined a corresponding sequence of goals whose satisfaction, one after another, cumulatively generated the performance **P**. For each length in the sequence, the local goal was to traverse the length from beginning to end according to the rules specified (see below) for the amplitude value of that length. In the biological interpretation, lengths with amplitudes of 1, −1, and 0 were to be respectively "ingested," "egested," and simply passed over in time. Although proceeding forward in time, the traversal of each length of amplitude −1 was easier to model as well as to interpret in the spatial dimension, as a retrograde motion of the position of the model back over the previous, identical length of amplitude 1, that is, as egestion of the seaweed strip that had just been ingested (see, e.g., Figures 6 and S2, and below). The global goal was then to maximize **P** by proceeding through as many lengths as possible in the entire simulation of some fixed duration, which implied proceeding through each length, on average, as fast as possible.

Extended in time, the sequence of goals constituted the time-continuous quantity $G(t)$, whose amplitude at any time, 1, −1, or 0 according to the amplitude value of the length then being traversed, represented the current local goal and was maintained until that goal was satisfied, that is, until that length had been traversed (see Figures 6, S2).

Extended in time in parallel with $G(t)$, the sequence of the true environment, in combination with feedback from the position of the model $P(t)$, also determined the true stimulus $S_t(t)$. If the amplitude value of the length currently being traversed was 1 or 0, then $S_t(t)$ was likewise 1 or 0, respectively. If the amplitude value of the length was −1, however, then $S_t(t)$ was −1 only if $P(t)$ was within 10 units of the position where the amplitude of the true environment had changed from 1 to −1; otherwise $S_t(t)$ was 1 (see Figures 6, S2). In the biological interpretation, the true stimulus thus derived only from the current point of contact with the environment: after a seaweed strip was found to be attached, it was felt to be "inedible" only over a short segment within 10 units of the point of attachment; otherwise each segment of strip, whether attached or free, was locally felt to be "edible."

As in Task 1, $S_p(t)$ was then constructed by modifying $S_t(t)$ over very short intervals with durations drawn randomly from an exponential distribution with a mean of 0.1 s. For each interval, a random number r was drawn from the uniform distribution on [0, 1]. If $r \le f$, the amplitude of $S_p(t)$ during the interval was set to the amplitude of $S_t(t)$ at the beginning of the interval, otherwise it was set to 0. [If $S_t(t)$ was itself 0, therefore, it remained unmodified.] In the biological interpretation, the perception of a true stimulus was thus intermittent. This procedure implied that in Task 2 the meaningful range of f was from 0, where $S_p(t)$ was 0 and the true stimulus was never perceived at all, to 1, where $S_p(t)$ was identical to $S_t(t)$ and the true stimulus was always fully perceived.

With $S_p(t)$ as the input $S(t)$, numerical integration of Equations S1 and S2 of the 2D model, or Equation S3 of the 1D model, starting from the initial conditions $B(0) = 0$, $M(0) = 0$, then produced $B(t)$ (see Figures 6, S2).

From $B(t)$, the position $P(t)$ was computed, according to different rules depending on whether the goal $G(t)$ was 1, −1, or 0. Overall, $P(t)$ was given by the equation

$$\frac{\mathrm{d}P(t)}{\mathrm{d}t} = \begin{cases} U\left(B(t)\right) & \text{if } G(t) = 1 \text{ or } -1 \\ 0 & \text{if } G(t) = 0 \end{cases}, \qquad \text{(S4)}$$

where U was the "utility" function that transformed the behavior B into its functional effect on

the position P, specifically the sigmoidal function

$$U(x) = \frac{2}{1 + \exp(x/0.05)} - 1 .$$

Equation S4 was integrated, starting from the initial condition $P(0) = 0$, along with Equations S1 and S2 or Equation S3. The sigmoidal utility function was chosen to enhance the functional effect of moderately positive (as well as negative) values of B to reproduce observations that suggest that moderately ingestive motor programs *in vitro* can correspond to quite strongly ingestive behavior in the intact animal. For example, while in Figure S1 $B(t)$ was still only moderately positive after three or four cycles of the CBI-2 stimulation, in intact animals, starting similarly from a neutral or intermediate state, highly effective ingestive movements can already be produced after the same number of cycles of the feeding behavior (e.g., [S12, S13]). The inclusion of a utility function different from the identity function in Task 2 can thus be seen as the beginning of explicit modeling of a "body" through which the interaction between the "brain" and the environment must pass [S14-S16]. With the utility function, B is reduced to describing only the motor programs produced by the CPG (from which B was modeled in the first place) while $B' = U(B)$ becomes the actual behavior of the animal. (In Task 1, $B' = B$.) More elaborate modeling of the body would then add dynamics for the transformation of $B(t)$ to $B'(t)$.

For further analysis (e.g., that in Figures S4 and S6), it is important to note that the utility function $U(x)$ is symmetrical about the origin, so that, in Equation S4, when $B(t) < 0$, $dP(t)/dt < 0$, when $B(t) = 0$, $dP(t)/dt = 0$, and when $B(t) > 0$, $dP(t)/dt > 0$.

Several additional rules accompanied Equation S4, to implement, most importantly, the progression of the simulation from one length of the true environment to the next. (1) $P(t)$ was not allowed to become negative or larger than l, the numerical value of the length of the true environment—if $G(t)$ was 1 or −1, the length of the seaweed strip—that was currently being traversed. (2) If $G(t)$ was 1 and $P(t)$ reached l, then the current seaweed strip had been completely ingested. The model moved on to the next length of the true environment. If that length had an amplitude of 0, then $P(t)$ was reset to 0. (3) If $G(t)$ was −1 and $P(t)$ reached 0, then the current (attached) seaweed strip had been completely egested. Again, the model moved on to the next length of the true environment. (4) Finally, if $G(t)$ was 0, then by Equation S4 $P(t)$ remained at 0 while all of the equations were integrated for an interval of time numerically equal to l. Together, Equation S4 and these rules produced $P(t)$ and a progression through the successive lengths of the environment of the kind seen in Figures 6 and S2.

The performance **P** was computed at the end of the entire simulation, as the sum of the lengths of amplitude 1 traversed minus the sum of the lengths of amplitude −1 traversed—that is, the net length of the "edible" free seaweed that was ingested in the simulation—divided by the duration of the simulation. Each simulation was run for 100,000 s, or 100 times longer than τ, whichever was longer. This was long enough to give a reasonably settled value of **P** at the end of the simulation. In the plots in Figure 5*C*, furthermore, the results of 8 independent simulations have been averaged together at each combination of τ and f, and likewise for each point in Figures S4*A* and S7*B* (red and green plots). (In Figure S5, the results of 100 simulations have been combined for a few special cases.) The variability remaining in **P** after averaging 8 simulations can be gauged from the error bars (SE, $n = 8$) in Figures S4*A* and S7*B* (red and green plots).

The absolute minimal performance **P** was 0, signifying no free seaweed ingested in the simulation. However, values of **P** very close to 0 could also come about in another way. A characteristic mode of behavior of the 2D model was that, if it failed to egest an attached seaweed strip completely, it would continue to oscillate back and forth on it, often essentially indefinitely (see Figure 6, and main text). In that case, no matter how much free seaweed had previously been ingested, **P** for the entire simulation would tend toward 0 as the simulation progressed further. To prevent this, we added a rule that, as in biological reality, the seaweed strip could break. Specifically, if $G(t)$ was 1 or −1, that is, if the model was ingesting or egesting a strip, the strip could break with a probability of 0.00001/s. The model then moved on to the next length of the true environment with amplitude 0, resetting $P(t)$ to 0, as if a free strip had just been ingested. The part of the broken strip that had been ingested, but not egested again, counted toward **P**. In principle, this rule ensured that although the oscillations on an attached strip would still continue for a long time, given the low break probability, they would not continue indefinitely, so that **P** would be low, but not 0, on average. In practice, since we ran multiple independent simulations of moderate duration rather than one very long simulation to compute **P**, the rule did not play a major role in this paper.

The maximal performance **P** that could be achieved in Task 2 can be readily calculated. For maximal performance, the model should proceed through the environment as quickly as possible, traversing each length of amplitude 1 with $B(t)$, and so $\mathrm{d}P(t)/\mathrm{d}t$, equal to 1, and each length of amplitude −1 with $B(t)$ and $\mathrm{d}P(t)/\mathrm{d}t$ equal to −1, thereby traversing each length in the amount of time equal to its numerical value l. For each length of amplitude 0, this already always happens by rule (4) accompanying Equation S4. With the statistics of the environment specified for Task 2, the lengths of amplitudes 1, −1, and 0 are present in the environment, on average, in the ratio 4:1:4, and each takes, on average, $l = \tau$ units of time to traverse. In those 9τ units of time, only the 3τ units of length of the 3 free seaweed strips (neglecting the rare strip breaks) count toward **P**, for a maximal average performance **P** of 1/3, at any τ. If no attached seaweed strips are encountered, the maximal average **P** rises to 1/2, and over a single free seaweed strip, the absolute maximal **P** is 1.

4. Analysis of 2D model in Task 2

The core of the behavior of the 2D model in the Task 2 environment is produced by the core system of the three dynamical variables, B, M, and P. In the full model and environment as described so far this system is driven by the highly stochastic S_p. The system can be tractably analyzed, however, if we assume that the noise that generates S_p from S_t does so on a time scale that can be considered instantaneous compared to the time scales of both S_t and B. If so, then, in any small interval of time in which $S_t(t) = 1$ and over which $B(t)$ does not change appreciably, the input from all of the subintervals with $S_p(t) = 1$, and all those with $S_p(t) = 0$, can be expressed as two instantaneous opposing forces, driving $B(t)$ toward 1 and 0 and weighted by f and $(1-f)$, respectively; and similarly for any small interval in which $S_t(t) = -1$. Rewriting Equation S1 in these terms, repeating Equation S2, and adding to Equation S4 its accompanying rule (1) yields the equations

$$\frac{\mathrm{d}B(t)}{\mathrm{d}t} = \begin{cases} f\left[k_x + k_{w1}M(t)\right]\left[1-B(t)\right] + \left[1-f\right]k_y\left[0-B(t)\right] & \text{if } S_\mathrm{t}(t)=1 \\ k_y\left[0-B(t)\right] & \text{if } S_\mathrm{t}(t)=0 \\ f\left[k_z + k_{w2}M(t)\right]\left[-1-B(t)\right] + \left[1-f\right]k_y\left[0-B(t)\right] & \text{if } S_\mathrm{t}(t)=-1 \end{cases}, \tag{S5}$$

$$\frac{\mathrm{d}M(t)}{\mathrm{d}t} = k_M\left[\max\left(B(t), 0\right) - M(t)\right], \tag{S6}$$

$$\frac{\mathrm{d}P(t)}{\mathrm{d}t} = \begin{cases} \left.\begin{matrix} \min\left(U\left(B(t)\right),0\right) & \text{if } P(t)=l \\ U\left(B(t)\right) & \text{if } 0<P(t)<l \\ \max\left(U\left(B(t)\right),0\right) & \text{if } P(t)=0 \end{matrix}\right\} \text{if } S_\mathrm{t}(t)=1 \text{ or } -1 \\ 0 \qquad\qquad\qquad\qquad\qquad\qquad\qquad \text{if } S_\mathrm{t}(t)=0 \end{cases}, \tag{S7}$$

where

$$U(x) = \frac{2}{1+\exp(x/0.05)} - 1$$

and $k_x = 0.02$, $k_y = 0.002$, $k_z = 0.00496$, $k_{w1} = 0.0368$, $k_{w2} = 2.93$, and $k_M = 0.01$, as before. These equations express the complete dynamics of the core system, when driven, now, by the true stimulus $S_t(t)$ as the input. They do not implement the feedback from $P(t)$ that in turn switches $S_t(t)$ from one value to the next (or any associated rule, such as the resetting of $P(t)$ to 0 after the ingestion of a free strip). The system is thus still nonautonomous; a fully autonomous system would require at least one more dynamical variable to represent the dynamics of S_t within the system itself (cf. [S17]). For convenience of visualization and analysis, however, we chose to retain the 3-dimensional system. We used it to study the dynamics over just one interval of constant $S_t(t)$, or piecewise over multiple intervals while switching the value of $S_t(t)$ by the same rules that were used with the full 2D model in Task 2 (see Figures 7, S3, S4, and S6).

It was simplest to integrate Equations S5-S7 numerically (e.g., in Figure 7), but we also obtained analytical solutions in certain cases. In particular, for the analysis in Figures S3 and S4*B*, where $M(t) = 0$, the solution of Equation S5 for constant $S_t(t) = 1$ and for constant $S_t(t) = 0$ is, respectively,

$$B(t) = \frac{k'_x}{k'_x + k'_y} - \left[\frac{k'_x}{k'_x + k'_y} - B(0) \right] \exp\left(-\left[k'_x + k'_y \right] t \right), \tag{S8}$$

where $k'_x \equiv f k_x$ and $k'_y \equiv [1 - f] k_y$, and

$$B(t) = B(0) \exp\left(-k_y t \right). \tag{S9}$$

And, for the analysis in Figure S6, the single, stable fixed point (B_∞, M_∞) to which all solutions ($B(t)$, $M(t)$) of Equations S5 and S6 tend as $t \to \infty$ with constant $S_t(t) = 1$ and with constant $S_t(t) = -1$ is, respectively

$$\left(B_\infty = \frac{k'_{w1} - k'_x - k'_y + \sqrt{4k'_{w1} k'_x + \left[k'_x + k'_y - k'_{w1} \right]^2}}{2k'_{w1}},\ M_\infty = B_\infty \right), \tag{S10}$$

where $k'_{w1} \equiv f k_{w1}$, and

$$\left(B_\infty = \frac{-k'_z}{k'_z + k'_y},\ M_\infty = 0 \right). \tag{S11}$$

With the values of k_x, k_y, k_z, and k_{w1} used, Equation S10 yields positive values of B_∞ and M_∞, and Equation S11 negative values of B_∞, for all $f > 0$.

How valid is the assumption of the analysis, that the noise in S_p can be considered instantaneous relative to the time scales of S_t and B, with respect to the simulations in this paper? In the simulations, the time scale of the noise in $S_p(t)$ was 0.1 s whereas τ, the scale of $S_t(t)$, ranged from 1 to 1000 units of length and so at least that many seconds, and $B(t)$ also usually changed relatively slowly (Figures 6, S2). The assumption thus appears to be sufficiently valid for the analysis to provide meaningful insight into the behavior of the system in the simulations. Nevertheless, several signs—the lack of local smoothness of $B(t)$ that is apparent, for example, in Figure 6, as compared to the perfectly smooth analytical relaxations in Figures 7 and S4*B1*, and likewise the variability of the successive oscillations in the right half of Figure 6—suggested that the structure of the noise in $S_p(t)$ did influence $B(t)$. Indeed, systematic alterations of the time scale or statistical structure of the noise altered $B(t)$: broadly speaking, slower noise speeded up, and faster noise slowed down, the relaxations of $B(t)$ (see also Section 5 below). Therefore the comparison between the analysis and the simulations must be considered to be semi-quantitative only.

5. Numerical integration methods

The differential equations was integrated numerically in *Mathematica* 5.1 (Wolfram Research, Champaign, IL). For numerical solutions of differential equations, *Mathematica* implements a nonstiff Adams method and a stiff Gear method, based on the LSODE routine [S18]. These implementations attempt to constrain the local error in a numerical solution of size x to be smaller than $10^{-a} + |x|10^{-p}$, where a is the number of digits of accuracy and p is the number of digits of precision. Standard settings were $a = 6$ and $p = 6$. Results with increased a and p were compared to confirm global accuracy.

In the Task 2 simulations, to implement the feedback from the position $P(t)$ back to the true environment and the true stimulus $S_t(t)$, the integration was performed over short intervals of time, typically $\tau/100$ s long, after each of which $P(t)$ was checked to see if it had passed one of the critical values, $P = 0$, $P = l$, or $P = l - 10$, that triggered progress on to the next length of the true environment or alteration of $S_t(t)$, as specified in Section 3.3. Although the integration intervals were short, nevertheless $P(t)$ was not known instantaneously, so that the progress was not triggered immediately when $P(t)$ reached a critical value, but only at some variable time up to $\tau/100$ s later. This is the reason why, for example, the sucessive oscillations of $P(t)$ in the right half of Figure 6 reach variable heights (in contrast to the comparable oscillations in Figure 7 of the analytical system, which was integrated quasi-continuously). The interval-by-interval integration scheme thus acted to increase the effective noise in the simulations beyond the noise explicitly present in $S_p(t)$.

6. Supplementary figure legends and discussion

6.1. Figure S1. Experimental data and fit of the 2D model

A: Experimental data from Figure 1 of Proekt et al. [S1]. Neuron CBI-2 was stimulated intracellularly to fire at 9 Hz over the duration of the protraction phase of the single feeding motor program that was elicited by each such period of stimulation. Across all repetitions of the CBI-2 stimulation in all experiments in *A* as well as in *C*, a reasonable single concensus value for the duration of the stimulation was 24 s. A 30-s period of rest, or in the second half of the experiment a defined longer period, was allowed before the next stimulation. The pattern of stimulation over the entire experiment is indicated by the small white blocks across the middle of the plot. Each block elicited one motor program whose mean frequencies of motor neuron B8 firing in the protraction and retraction phases were measured. The grey bars show the means ± SE of the protraction (downward bars) and retraction (upward bars) frequencies from n = 7 experiments. Before each experiment in *A* as well as *B* and *C*, the preparation was kept for a prolonged period unstimulated and quiescent.

B: Experimental data from Figure 1 of Proekt et al. [S1]. As in *A* except with extracellular stimulation of EN at 2 Hz for 2 min with the amplitude of the stimulation adjusted to elicit a number of motor programs during the 2-min period, then with brief periods of EN stimulation to elicit single programs. The pattern of stimulation over the entire experiment is indicated by the small black blocks across the middle of the plot. n = 6 experiments.

C: Experimental data from Figure 2 of Proekt et al. [S1] and additional data from the same experiments. As in *A* and *B*, but combining the stimulation of CBI-2 and EN in the pattern shown by the pattern of the white and black blocks across the middle of the plot. In these experiments, EN was stimulated for 5 min and elicited 8.7 ± 0.7 motor programs during that time (mean ± SE, n = 13 experiments). Thus a reasonable single concensus value for the duration of the EN stimulation that elicited a single program was 30 s. In *B* and *C*, all of the bars corresponding to the EN stimulation are therefore 30 s long, except the last EN bar in *C* which is 60 s long to pool a sufficiently large number of programs. Each bar in *C* contains n = 6-18 programs from 13 experiments.

The solid red curves show the best fit of the 2D model with the stimulation patterns shown. The dashed red curve in *C* shows the behavior of the model if, after the first 30 s of EN stimulation, the stimulation is switched back to the same CBI-2 pattern as at the beginning of *C*.

For further details and explanation of *D* see Section 1.

6.2. Figure S2. Low performance of the 2D model in Task 2 in a short environment

Shown is a representative simulation at $\tau = 3, f = 0.1$, presented in the same manner as the simulation at $\tau = 200, f = 0.1$ in Figure 6. As indicated by the black coloring of the whole length of the attached seaweed strips 17 and 20, these strips, shorter than the critical length of 10, once they are found to be attached, are felt to be inedible over their whole length [$S_t(t) = -1$]. No goal-driven egestion therefore needs to be performed: the behavior is stimulus-driven through the entire simulation [$G(t) = S_t(t)$ throughout]. In contrast to Figure 6, the model is therefore guaranteed to egest each attached strip completely and to continue to make progress through the simulation. However, the progress is relatively slow. It takes many of these short strips before their ingestive stimulus integrates to raise $B(t)$, and so the rate of ingestion $dP(t)/dt$,

to even modest positive values. And, on average, only three free strips will be encountered before $B(t)$ is reset to a negative value again by the egestive stimulus of an attached strip (the initial run of 16 free strips in this simulation is rare). It then takes an equally long time for $B(t)$ to recover sufficiently to begin the ingestion of the next strip. The egestion of a strip, even when successful, thus imposes a considerable delay penalty on the ingestion of the next strip. All of these phenomena occur in longer environments too, but here the time penalties they impose are much larger relative to the length of the strips that are eaten. Performance in this region of the environmental space ("*a*" in Figure 5*C*, left) is consequently low.

6.3. Figure S3. Complete analysis of the shape of the region of high performance of the 2D model in the Task 2 environment, region "*b*" in Figure 5*C*, left.

(1) "Full 2D model" (black circles). All of the values of the performance, **P**, that were used to generate Figure 5*C*, left, are plotted as a function of (log) τf.

(2) "Analytical system" (blue circles). The corresponding plot of **P**, given by 3τ/(limit cycle period) for a "successful" limit cycle and 0 for a "failed" limit cycle, computed from all of the limit cycle period values that were used to generate Figure 7*B*.

Both plots show similar behavior. Above some value of τf—rather sharply above $\tau f \approx 17$ in the case of the full 2D model—**P** collapses due to the failure to completely egest attached seaweed strips, as described in the main text. Just below this critical value of τf, **P** attains its highest values, not essentially different from its highest possible value of 1/3 (horizontal dashed line; see Section 3.3). This represents the leading edge of the high-performance region "*b*", facing region "*c*", in Figure 5*C*, left. As τf decreases further below the critical value, away from the leading edge of the region "*b*" toward region "*a*" in Figure 5*C*, left, **P** decreases. By the definition of **P** (see Section 3.3), this must be because the model progresses at a relatively slower rate through the simulation, which in turn, since the rate dP/dt is driven by the behavior B, is most likely because the absolute amplitude of B decreases. Both are in fact observed in the simulations (see, e.g., Figures 7*B* and S2). Throughout all this, **P**, the rate of progress, and the amplitude of B are determined, not by τ or f separately, but by the product τf. Thus, τ and f trade off, large τ and small f having the same effect as small τ and large f. This explains the conspicuously curved shape of the high-performance region in Figure 5*C*, left.

The trade-off between τ and f suggests that the essential determinant is the total amount of nonzero perceived stimulus S_p that the slow dynamics of B integrate from each seaweed strip of length τ. However, for this integrated S_p to determine the amplitude of B, B must be far from the steady state. The slow dynamics of B integrate S_p also from one strip to the next (Figures 6 and S2), so that over a succession of free strips B will eventually build up to a large positive amplitude (although, in a nonlinear system, not necessarily to exactly the same amplitude [S19]) whether τ is large or small, so that in the steady state the amplitude of B will become relatively insensitive to τ. A periodic resetting of B away from the steady state is provided, however, by the interposed attached strips (Figures 6 and S2). To clarify the significance of this resetting, we performed the following numerical analysis.

(3) "Analysis" (red circles). We used the analytical system as in (2), except with $M(t) = 0$, a simplification that allows analytical solutions to be written for $B(t)$ when driven by constant $S_t(t) = 1$ or 0, given by Equations S8 and S9, respectively, in Section 4. For each specific τ and f, we constructed a longer waveform of $B(t)$ by joining end-to-end segments of Equation S8

alternating with segments of Equation S9, all segments having length τ, where the starting value $B(0)$ of each segment was the final value $B(\tau)$ of the previous segment; the first segment, of Equation S8, started with $B(0) = 0$. The waveform rose relatively rapidly during each of the segments of Equation S8 and fell slowly during those of Equation S9, so that cumulatively it rose much like the waveform of $B(t)$ following each resetting by an attached strip in the simulations (Figures 6 and S2; see also Figure 7*C*). We then used Equation S7 to compute the mean rate of ingestion $dP(t)/dt$ over the segments of Equation S8, which, when divided by 3 to scale it to the maximal value of 1/3, provided a measure of the performance **P**.

We then varied the number of segments comprising the waveform. In the simulations there are, on average, 3 free strips before a resetting by an attached strip. Plotted here is therefore the performance of waveforms with 3 segments of Equation S8, over the same range of values of τ and f as in (1). With 3 segments, clearly, the essential determinant of performance is still, as in the simulations, the product τf. As the number of segments increased further, however, τ and f began to have separate effects (not shown), until, in the steady state after a very large number of segments (or as solved analytically [S16, S19]), τ and f no longer traded off at all. The control by the product τf is, therefore, a transient phenomenon. The slow dynamics of B accumulate the total amount of the perceived stimulus S_p from all of the seaweed between one resetting by an attached strip and the next, and determine accordingly the rate of progress through the simulation and the performance **P**, but this can only happen if the attached strips occur frequently enough that the system is operating most of the time in the transient, rather than the steady-state, regime (cf. [S20]).

The analysis so far explains the behavior of the system below the critical value of $\tau f \approx 17$, that is, in the "successful" dynamical mode of the system (see main text). The fact that the transition to the "failed" mode is also determined by the product τf, occurring at a constant $\tau f \approx 17$, has a different, but related, reason. It can be roughly analyzed as follows. The attached strip resets B, in fact, all the way to negative values that drive the egestion of the strip (Figure 6). During the goal-driven phase of the egestion, B then relaxes back in the positive direction as the slow dynamics integrate S_p as described above. The egestion terminates, at the latest, when B reaches zero. The time that it takes to reach this point therefore determines the maximal length of the attached strip that can be successfully egested—the critical value of τ (see Figure S4*B*). But, according to the analysis above, B relaxes faster, and so reaches $B = 0$ sooner, as f, the density of S_p, increases. Thus the critical τ decreases as f increases, and the critical τf remains, roughly, a constant.

6.4. Figure S4. The slow dynamics of the behavior B*, rather than the decay of the memory* M*, determine the longest seaweed strip that can be egested*

A: Performance of the full 2D model in Task 2 simulations, as in Figures 5, 6, and S2, plotted over τ ranging from 1 to 300, with $f = 0.1$. Each point is the mean ± SE of 8 simulations. *A1*: Simulations with k_M, the rate constant of the memory, equal to 0.01 as in the standard 2D model and then 10-fold larger or smaller. *A2*: Simulations with k_x, the principal rate constant that governs the slow evolution of B in the ingestive direction, equal to 0.02 as in the standard 2D model and then 10-fold larger or smaller. The changes in k_x shift the peak of high performance, and the point of collapse of the performance due to the failure to completely egest longer attached seaweed strips, along the τ-axis correspondingly, whereas the changes in k_M have

little effect.

B: Analysis of the control by k_x of the duration of the goal-driven phase of egestion and hence the length of seaweed that is egested. *B1*: During the initial, stimulus-driven phase of the egestion of an attached strip, $S_t(t) = -1$, $B(t)$ is rapidly reset to a negative value, so therefore is $dP(t)/dt$, and the egestion begins (Figure 6). Then, after 10 units of length have been egested, $S_t(t) = 1$ and the egestion becomes goal-driven (cartoon at top). $B(t)$ slowly relaxes back from the negative values to which it had been reset, and the egestion continues as long as $B(t)$, and so $dP(t)/dt$, remains negative. In the analytical system (Section 4) with $M(t) = 0$, these relaxations of $B(t)$ are simple exponentials, given by Equation S8. The middle plot shows three representative relaxations, starting in each case from $B(0) = -0.5$, a reasonable value to which $B(t)$ might be reset during the stimulus-driven phase of the egestion (see Figure 6), with $f = 0.1$, and with the three values of k_x in *A2*. The bottom plot then shows the three relaxations integrated through Equation S7, starting from $P(0) = 0$, to give $P(t)$, the length egested. The maximal length that can be egested is the (absolute) value of $P(t)$ when $B(t)$ reaches zero, when the seaweed strip begins to be ingested again; if the strip is longer, it fails to be egested completely. *B2*: The maximal length of seaweed that can be egested before failure as a function of (log) k_x, determined as in *B1* with the 10 units always egested in the stimulus-driven phase added. The three colored dots correspond to the three cases in *B1*.

6.5. Figure S5. Prediction of the environment by the 2D model in Task 2

In each Task 2 simulation, the true environment consists of various lengths of free and attached seaweed. In response, the model produces various durations and strengths of ingestive and egestive behavior. As in Task 1 but length-by-length rather than instantaneously, these responses can be interpreted as the model's best prediction, as expressed in the behavior, of what the true environment is.

Here, we performed 100 simulations, as in Figures 5 and 6, in each environment, and compiled:

(1) The distributions of the lengths of the true environment with stimulus amplitudes of 1 and −1, essentially the distributions of the lengths of the free and attached seaweed strips but in a form suitable for comparison with (2) and (3) (see Section 3.3). Only the first-order statistics were considered here.

(2) The distributions of the durations of the true ingestive and egestive stimuli, that is, of the intervals in which $S_t(t)$ was 1 and −1, respectively. These were different from (1) because they depended also on the feedback from the behavior of the model.

(3) The distributions of the durations of the ingestive and egestive responses to the seaweed, that is, of the intervals in which the rate of ingestion or egestion $dP(t)/dt$ was positive and negative, respectively, when seaweed was actually present, that is, when $S_t(t)$ was not zero. We also computed the strength of each response, the mean $dP(t)/dt$ of each such interval. In terms of the prediction of the environment, the duration of the response can be interpreted as the model's estimate of the length of the seaweed strip, the sign of the response as an estimate of the free or attached quality of the strip, and the strength of the response as the degree of confidence in those estimates, a readiness to commit to them in action.

In *A1-5*, for five representative environments, the distributions (1)-(3), both ingestive (green) and egestive (red), are plotted at the top. The vertical scale of each plot indicates the number of intervals (events) in all 100 simulations. The 2-dimensional plot underneath is then a

probability density map of the response strength against the response duration, color-coded as indicated at bottom left, with a maximal probability density (most red color) of 0.0021 in *A1*, 0.0071 in *A2*, 0.0020 in *A3*, 0.0071 in *A4*, and 0.0016 in *A5*.

B, top, shows the locations of the five environments in *A* on a map of the performance **P**, reproduced from Figure 5*C*, left, and extended to $\tau = 500$. *B*, bottom, plots the mean ± standard deviation of the durations of the ingestive (green) and egestive (red) responses—that is, of the distributions (3), compiled from 100 simulations in each environment as in *A*—across a range of environments varying in τ, all at $f = 0.1$. The ingestive and egestive plots are slightly offset relative to each other for clarity. By our interpretation, these plots represent the model's estimate of the length of the environment. The (nominal) true length of the environment is indicated by the grey wedge.

We find that the model predicts the true environment correctly when τ is of the order of 70-100 at $f = 0.1$, and at progressively smaller values of τ as f increases. Indeed, the region where the environment is predicted correctly is more or less the region where the performance **P** is high. In this region, the model correctly estimates, by the durations of its responses, the true overall lengths of the seaweed strips (*B*, bottom, and *A2*, *A3*), rather than merely reproducing the durations of the local stimuli S_t, which, particularly in the egestive case, are much shorter [compare the distributions (1)-(3) in *A2* and *A3*]. Thus, from the local S_t (furthermore available only in its noisy form S_p) the model is able to synthesize, by the goal-driven dynamical mechanism, the correct estimate of the global true length of the environment. (That the true length and the response distributions match on average does not guarantee that they do so at the level of each individual strip, but substantial matching also at this level is implied by the high performance **P**.) In this region, the model estimates correctly also the ratio of the free and attached seaweed strips [compare the green and red distributions (1) and (3) in *A2* and *A3*]. Finally, it responds to these estimates, in which it can thus be said to have high confidence, with very strong responses (note the locations of the hot spots in the density plots in *A2* and *A3*).

With values of τ smaller than those in the high-performance region—in shorter environments—the model responds slowly (Figure S2) and so estimates the seaweed strips, furthermore with lower confidence, to be significantly longer than they truly are (*A1*). With large values of τ—in long environments—the model spends much of its time in the cycles of failed egestion (Figures 6, 7, and S6), in which it estimates the environment completely incorrectly as being densely packed with equal numbers of short free and attached strips (*A4* and *A5*).

6.6. Figure S6. More detailed dynamical analysis of the success or failure of the ingestion and egestion of seaweed strips of different lengths

This analysis, like that in Figure 7, was performed with simulations, supplemented by some analytical results, in the analytical system (Section 4) in which the state point is driven by the true stimulus S_t through the 3-dimensional space spanned by the behavior B, ranging from -1 to 1, the memory M, ranging from 0 to 1, and the position P, ranging from 0 to l, the length of the seaweed strip. Each of the two plots in *A* shows this space for $l = 150$. Whether ingestion of the strip [that is, the behavior when the goal $G(t) = 1$] succeeds or fails then substantially reduces to the question, from which combinations of B and M in the plane $P = 0$ does the system reach the plane $P = l$ (success) or, conversely, returns to the plane $P = 0$ (failure)? Similarly, whether egestion of the strip [the behavior when $G(t) = -1$] succeeds or fails reduces to the question,

from which combinations of B and M in the plane $P = l$ does the system reach the plane $P = 0$ (success) or, conversely, returns to the plane $P = l$ (failure)?

With success and failure defined thus, each of the colored planes in *B* maps, for all combinations of B and M, the success (green) or failure (never occurs) of the ingestion of a free or attached strip (middle column), the success (red) or failure (blue) of the subsequent egestion of an attached strip (right column), and the success (red) or failure (blue) of the ingestion followed by the egestion, that is, of the complete encounter with an attached strip (left column). These maps are shown for selected values of the strip length l (rows) in the intermediate range $80 \le l \le 260$. For all $l < 80$, the results are the same as for $l = 80$, and for all $l > 260$, the same as for $l = 260$. All of these maps are plotted for $f = 0.1$; other values of f give the same qualitative, although a different quantitative, picture.

As the solid green color of all of the maps in the middle column indicates, ingestion always succeeds, no matter what combination of B and M the system starts from, no matter how long the seaweed strip is. This can in fact be demonstrated analytically. Ingestion is always stimulus-driven, with $S_t(t) = 1$ throughout. With $S_t(t) = 1$, from a basin of attraction that comprises all values of B and M, all solutions ($B(t)$, $M(t)$) of Equations S5 and S6 tend as $t \to \infty$ toward a single, stable fixed point, (B_∞, M_∞), where B_∞ is positive for all $f > 0$ (Equation S10). Thus $B(t)$ always eventually becomes positive, so therefore, by Equation S7, does $\mathrm{d}P(t)/\mathrm{d}t$, and $P(t)$ continues to increase until $P = l$, no matter how large l is. Furthermore, according to Equation S5, $\mathrm{d}B(t)/\mathrm{d}t > 0$ if $B(t) \le 0$; thus, when $B(t)$ is not positive, it relaxes in the positive direction monotonically, and once it becomes positive, it remains so. By the symmetry of the function $U(x)$ in Equation S7, $\mathrm{d}P(t)/\mathrm{d}t$ has exactly the same properties. This guarantees that, although the system often first moves within the plane $P = 0$ (see, e.g., Figure 7*A1*), once it leaves it does not return, but reaches $P = l$ (e.g., the green trajectories in *A*).

Analogous arguments apply to stimulus-driven egestion, which occurs until 10 units of length of the attached seaweed strip have been egested, that is, in the top layer of the analytical space where $l - 10 \le P \le l$, or throughout the space if $l \le 10$. With $S_t(t) = -1$, B_∞ is negative (Equation S11), so that $B(t)$ always eventually becomes negative and $P(t)$ leaves the plane $P = l$ to reach $P = l - 10$, or $P = 0$ if $l \le 10$. The stimulus-driven phase of egestion thus always succeeds, too.

As the red color of the maps in the right column indicates, however, egestion always succeeds not merely with strips 10 units long, but with strips up to at least 80 units long, whose complete egestion requires at least 70 additional units of goal-driven egestion, with $S_t(t) = 1$, beyond the plane $P = l - 10$. Since that plane clearly contains a substantial region, at least that where B is positive, from which the system would return to $P = l$ with $S_t(t) = 1$, this must mean that no trajectories from the plane $P = l$ intersect the plane $P = l - 10$ in that region. Rather, thanks to the fast dynamics that reset $B(t)$ negative during the stimulus-driven phase of the egestion (Figures 6 and 7*C*), the trajectories from all combinations of B and M in the plane $P = l$ pass through the plane $P = l - 10$ with $B(t)$ negative enough that, although $B(t)$ then slowly relaxes in the positive direction as analyzed above in ingestion (see also Figure S4*B*), the trajectories all succeed in reaching $P = 0$ before $B(t)$ reaches zero (e.g., the red trajectory in *A*).

As the seaweed strip is made longer than ~80 units, however, this mechanism begins to fail (blue color in the right column). From some combinations of B and M in the plane $P = l$, the trajectories fail to reach $P = 0$ before $B(t)$ reaches zero, whereupon, for the same reasons as analyzed in ingestion, the trajectories must necessarily return to $P = l$ (e.g., the blue trajectory in

A). With intermediate strip lengths, the successful and the failed combinations of *B* and *M* coexist in the plane $P = l$, until, when the strip becomes longer than ~260 units, egestion always fails.

The left column shows the success or failure of the complete cycle of ingestion, followed by egestion, of the attached seaweed strip—a functional composite of the middle and right columns. These maps show the combinations of *B* and *M* in the plane $P = 0$ from which the system succeeds in reaching $P = l$ *and* returning to $P = 0$ (red), or fails in one of these two phases (blue). Since the ingestion always succeeds, any failure must occur in the egestion. Thus, all combinations of *B* and *M* in a blue region in the left column must map, through the middle column, to a blue region in the right column, and likewise for the red regions. The black and white circles and arrows across the columns show examples of such mappings. Again, with strips of intermediate length, success and failure coexist. The two plots in *A* show successful (top) and failed (bottom) trajectories, corresponding to the two mappings indicated, from two different combinations of *B* and *M* at the same $l = 150$.

As defined above, success and failure refer to the first attempt only. Ingestion and stimulus-driven egestion always succeed at the first attempt. When goal-driven egestion fails, however, the system returns to $P = l$ and makes another attempt (Figures 6, 7*C*, and the blue trajectory in *A*). Does a second, or later, attempt to egest ever succeed when the previous attempts have failed? When the trajectories are perturbed by noise, this can certainly happen (see below). Without noise, however, there are few, if any, locations in the analytical space where a failed attempt is followed by a successful one. A failed egestion maps a location in a blue region in the right column back onto the same map. Success following failure would be seen as a mapping from a blue to a red location. Instead, blue locations map to other blue locations, furthermore in such a way that the paths through the blue region all converge to a single blue location. Thus, in the example shown for $l = 170$, trajectories from the entire plane $P = 0$ (sampled by the grid of white circles in the left column) reach a much smaller region of the plane $P = l$ (small white circles in the right column), from which they then keep returning to the plane $P = l$ in such a way that they all converge to a single location (large white circle) that represents the intersection with the plane $P = l$ of a single, identical limit cycle (see *A*, bottom, and Figure 7*C*). Without noise, therefore, the system becomes trapped in the cycles of failed egestion.

As this analysis shows, the trajectories from the planes $P = 0$ and $P = l$ flow through some regions of the 3-dimensional analytical space and avoid others. What happens if a perturbation, or the noise that is present in the full 2D model, displaces the system into one of the latter regions? Although it is not usually visited, in such a region there is nevertheless a flow that, by the analysis, will carry the trajectory to either $P = 0$ or $P = l$. With constant $S_t(t)$, cycles that do not intersect either $P = 0$ or l are not possible. Once at $P = 0$ or l, the system will continue as analyzed. A perturbation may thus cause even ingestion or stimulus-driven egestion to fail; however, the next attempt will succeed (unless prevented by further perturbation). In the case of goal-driven egestion, if the seaweed strip is of intermediate length so that both success and failure are possible, the perturbation may switch the system from failure to success or vice versa—in which case the failure will then be repeated until reversed by further perturbation—as seen, for example, in Figure 7*C*.

6.7. Figure S7. Added variability enhances the performance of the 2D model in Task 2 simulations

Variability was added to the behavior *B* by offsetting $B(t)$ every 10 s of the simulation by a value drawn randomly from a Gaussian distribution with mean 0 and a given standard deviation, but keeping $B(t)$ in the range [−1, 1].

A: Performance mapped over the environmental space, as in Figure 5*C*, left, with the standard deviation of the variability set to 0 (this is therefore the same map as in Figure 5*C*, left), 0.1, 0.3, and 1.0.

B: Performance at certain locations in the environmental space, those marked in *A*, left, as a function of the standard deviation of the variability. Each point is the mean ± SE of 8 (red and green plots) or 96 (black plot) simulations.

We can see that where the performance is high already without the variability, the variability decreases it (red plot in *B*, red circle in *A*, left), but in many locations where the performance is low, the variability, up to a certain point, increases it (green plot in *B*, green circle in *A*, left). The latter is found, too, when the performance is averaged over a significant region of the environmental space (black plot in *B*, box in *A*, left). The overall conclusion, also suggested by the maps in *A*, is that a certain level of variability broadens the region of high performance in the environmental space.

7. Supplementary references

S1. Proekt A, Brezina V, Weiss KR (2004) Dynamical basis of intentions and expectations in a simple neuronal network. Proc Natl Acad Sci USA 101: 9447-9452.

S2. Morton DW, Chiel HJ (1993) In vivo buccal nerve activity that distinguishes ingestion from rejection can be used to predict behavioral transitions in *Aplysia*. J Comp Physiol A 172: 17-32.

S3. Morton DW, Chiel HJ (1993) The timing of activity in motor neurons that produce radula movements distinguishes ingestion from rejection in *Aplysia*. J Comp Physiol A 173: 519-536.

S4. Hurwitz I, Neustadter D, Morton DW, Chiel HJ, Susswein AJ (1996) Activity patterns of the B31/B32 pattern initiators innervating the I2 muscle of the buccal mass during normal feeding movements in *Aplysia californica*. J Neurophysiol 75: 1309-1326.

S5. Nargeot R, Baxter DA, Byrne JH (1997) Contingent-dependent enhancement of rhythmic motor patterns: an *in vitro* analog of operant conditioning. J Neurosci 17: 8093-8105.

S6. Kabotyanski EA, Baxter DA, Byrne JH (1998) Identification and characterization of catecholaminergic neuron B65, which initiates and modifies patterned activity in the buccal ganglia of *Aplysia*. J Neurophysiol 79: 605-621.

S7. Jing J, Weiss KR (2001) Neural mechanisms of motor program switching in *Aplysia*. J Neurosci 21: 7349-7362.

S8. Jing J, Weiss KR (2001) Interneuronal basis of the generation of related but distinct motor programs in *Aplysia*: implications for current neuronal models of vertebrate intralimb coordination. J Neurosci 22: 6228-6238.

S9. Morgan PT, Jing J, Vilim FS, Weiss KR (2002) Interneuronal and peptidergic control of motor pattern switching in *Aplysia*. J Neurophysiol 87: 49-61.

S10. Orekhova IV, Jing J, Brezina V, DiCaprio RA, Weiss KR, Cropper EC (2001) Sonometric measurements of motor-neuron-evoked movements of an internal feeding structure (the radula) in *Aplysia*. J Neurophysiol 86: 1057-1061.

S11. Proekt A, Jing J, Weiss KR (2007) Multiple contributions of an input-representing neuron to the dynamics of the *Aplysia* feeding network. J Neurophysiol 97: 3046-3056.

S12. Weiss KR, Koch UT, Koester J, Rosen SC, Kupfermann I (1982) The role of arousal in modulating feeding behavior of *Aplysia*: neural and behavioral studies. In: Hoebel BG, Novin D, editors. *The Neural Basis of Feeding and Reward*. Brunswick, ME: Haer Institute, pp. 25-57.

S13. Rosen SC, Weiss KR, Goldstein RS, Kupfermann I (1989) The role of a modulatory neuron in feeding and satiation in *Aplysia*: effects of lesioning of the serotonergic metacerebral cells. J Neurosci 9: 1562-1578.

S14. Chiel HJ, Beer RD (1997) The brain has a body: adaptive behavior emerges from interactions of nervous system, body and environment. Trends Neurosci 20: 553-557.

S15. Clark A (1997) *Being There*. Cambridge, MA: MIT Press.

S16. Brezina V, Orekhova IV, Weiss KR (2000) The neuromuscular transform: the dynamic, nonlinear link between motor neuron firing patterns and muscle contraction in rhythmic behaviors. J Neurophysiol 83: 207-231.

S17. Beer RD (1997) The dynamics of adaptive behavior: a research program. Robot Auton Syst 20: 257-289.

S18. Wolfram S (1999) *The Mathematica Book* (4th ed). Champaign, IL: Wolfram Media.

S19. Brezina V, Orekhova IV, Weiss KR (1997) Control of time-dependent biological processes by temporally patterned input. Proc Natl Acad Sci USA 94: 10444-10449.

S20. Morris LG, Hooper SL (1997) Muscle response to changing neuronal input in the lobster (*Panulirus interruptus*) stomatogastric system: spike number- versus spike frequency-dependent domains. J Neurosci 17: 5956-5971.
