# Supplementary figures and images for: Predicting Adaptive Behavior in the Environment from Central Nervous System Dynamics
Source: PLoS One. 2008 Nov 7;3(11):e3678. doi: 10.1371/journal.pone.0003678 (PMC2576442; doi:10.1371/journal.pone.0003678)

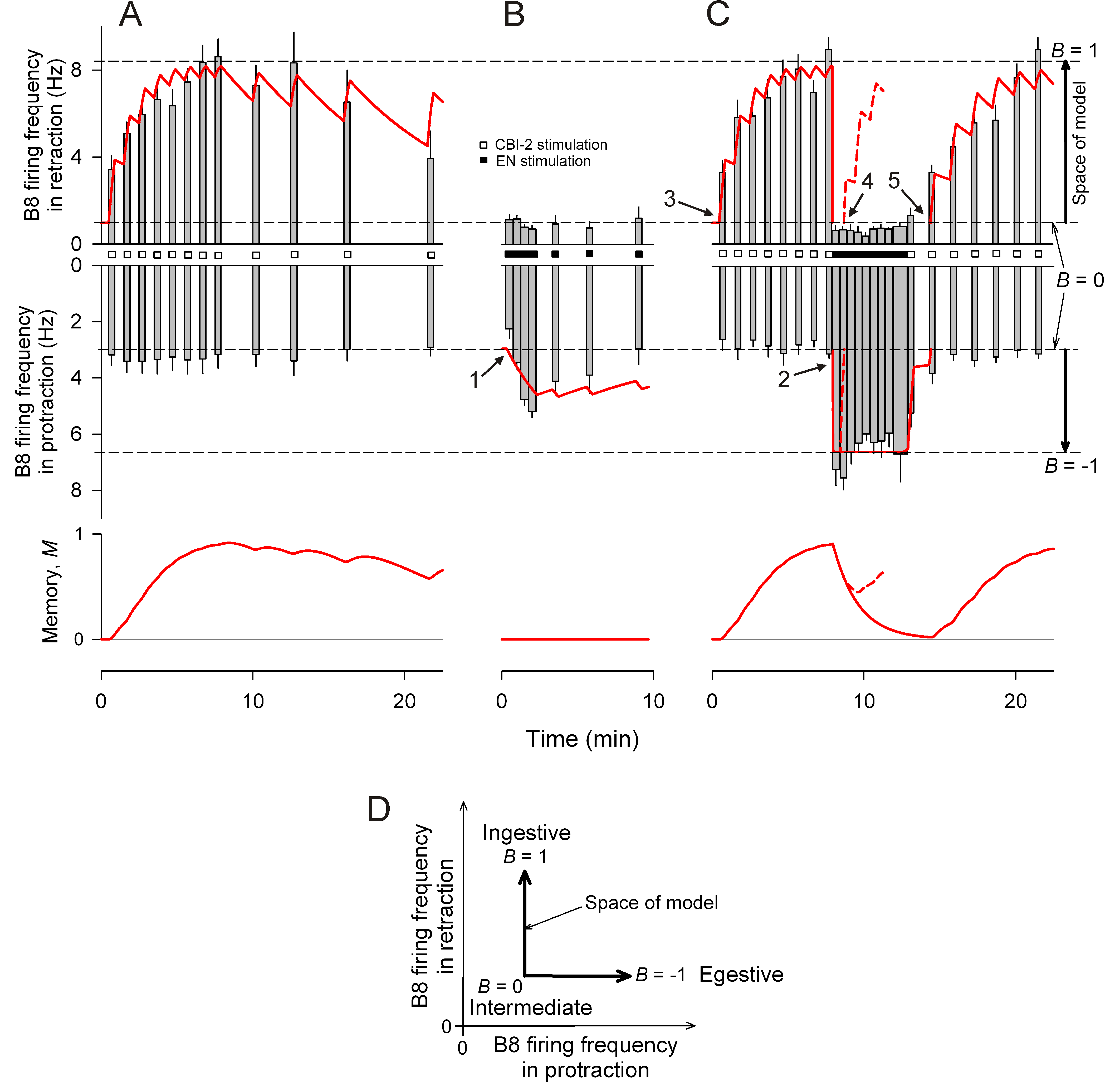

Supplement: Figure S1 — Experimental data and fit of the 2D model. (1.24 MB TIF) [file pone.0003678.s002.tif]

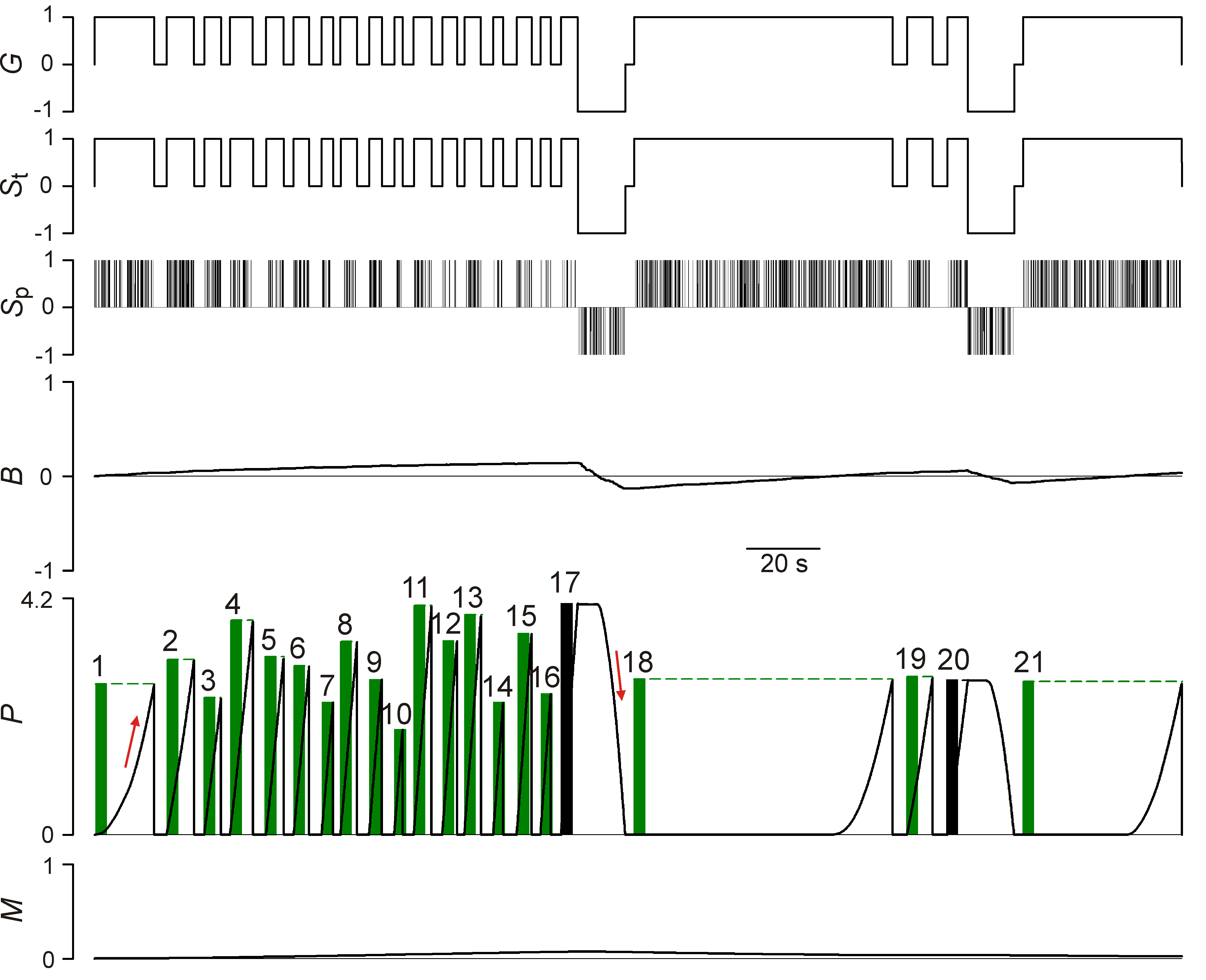

Supplement: Figure S2 — Low performance of the 2D model in Task 2 in a short environment. (1.08 MB TIF) [file pone.0003678.s003.tif]

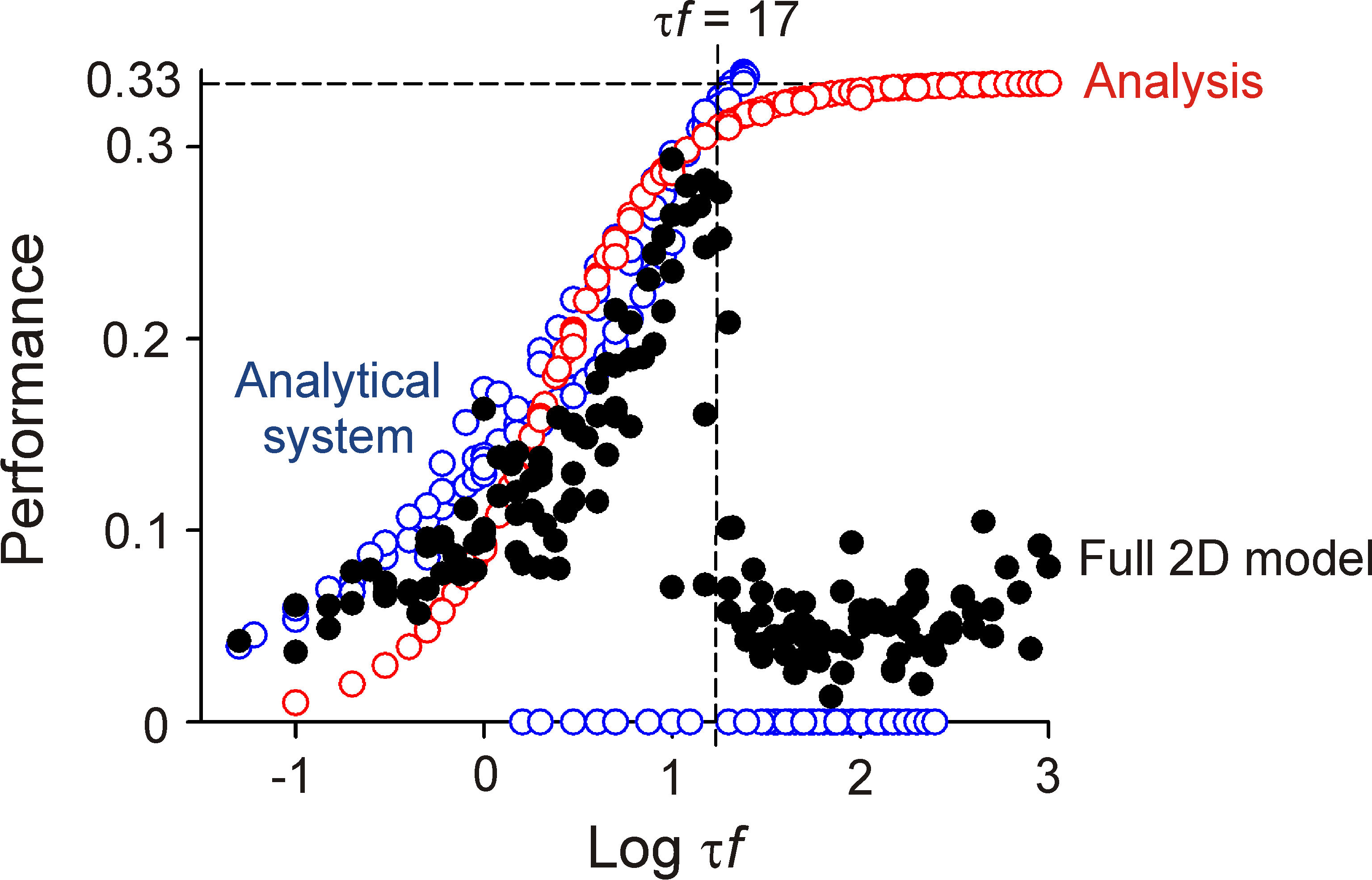

Supplement: Figure S3 — Complete analysis of the shape of the region of high performance of the 2D model in the Task 2 environment. (0.33 MB TIF) [file pone.0003678.s004.tif]

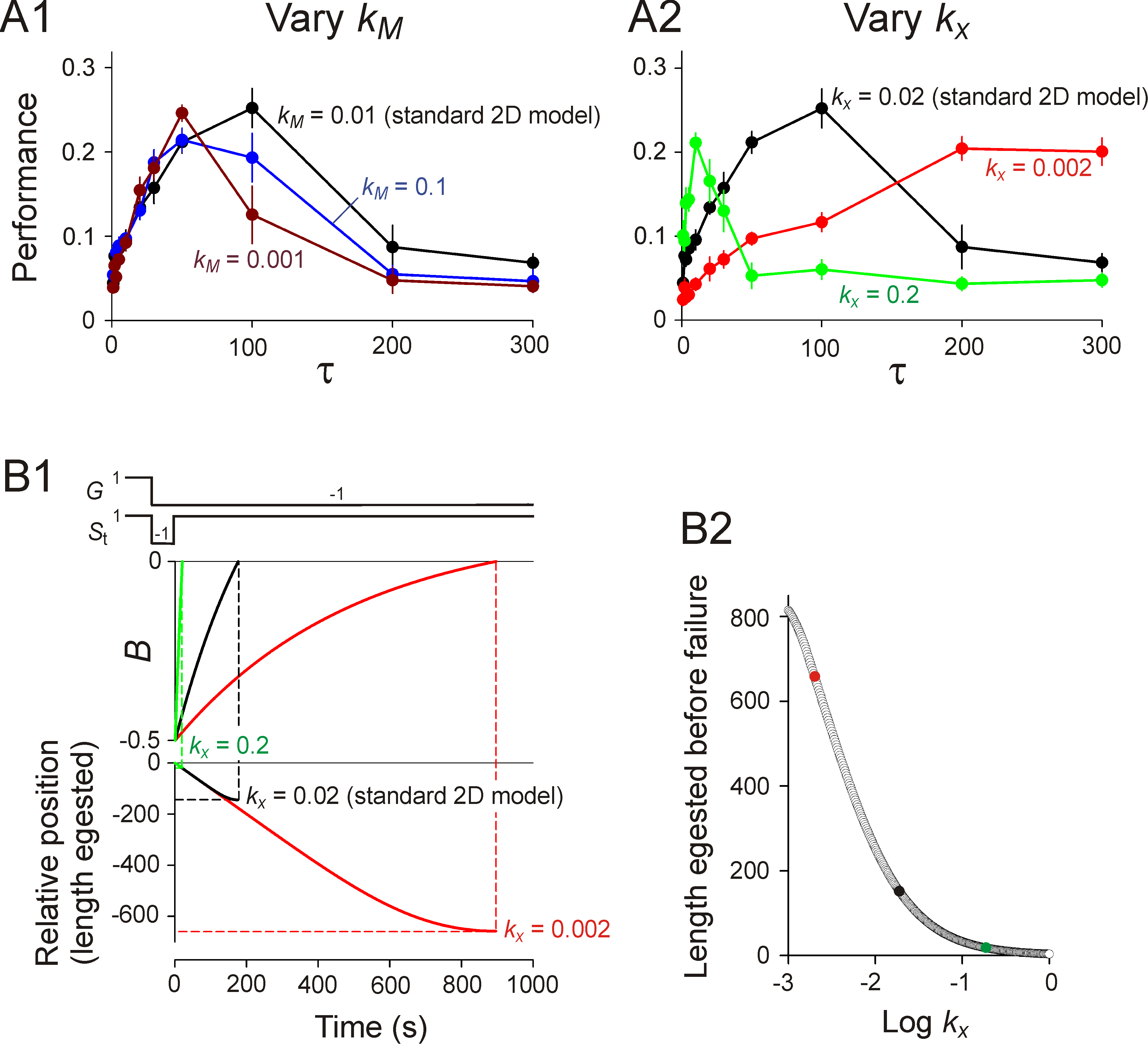

Supplement: Figure S4 — The slow dynamics of the behavior B, rather than the decay of the memory M, determine the longest seaweed strip that can be egested. (0.92 MB TIF) [file pone.0003678.s005.tif]

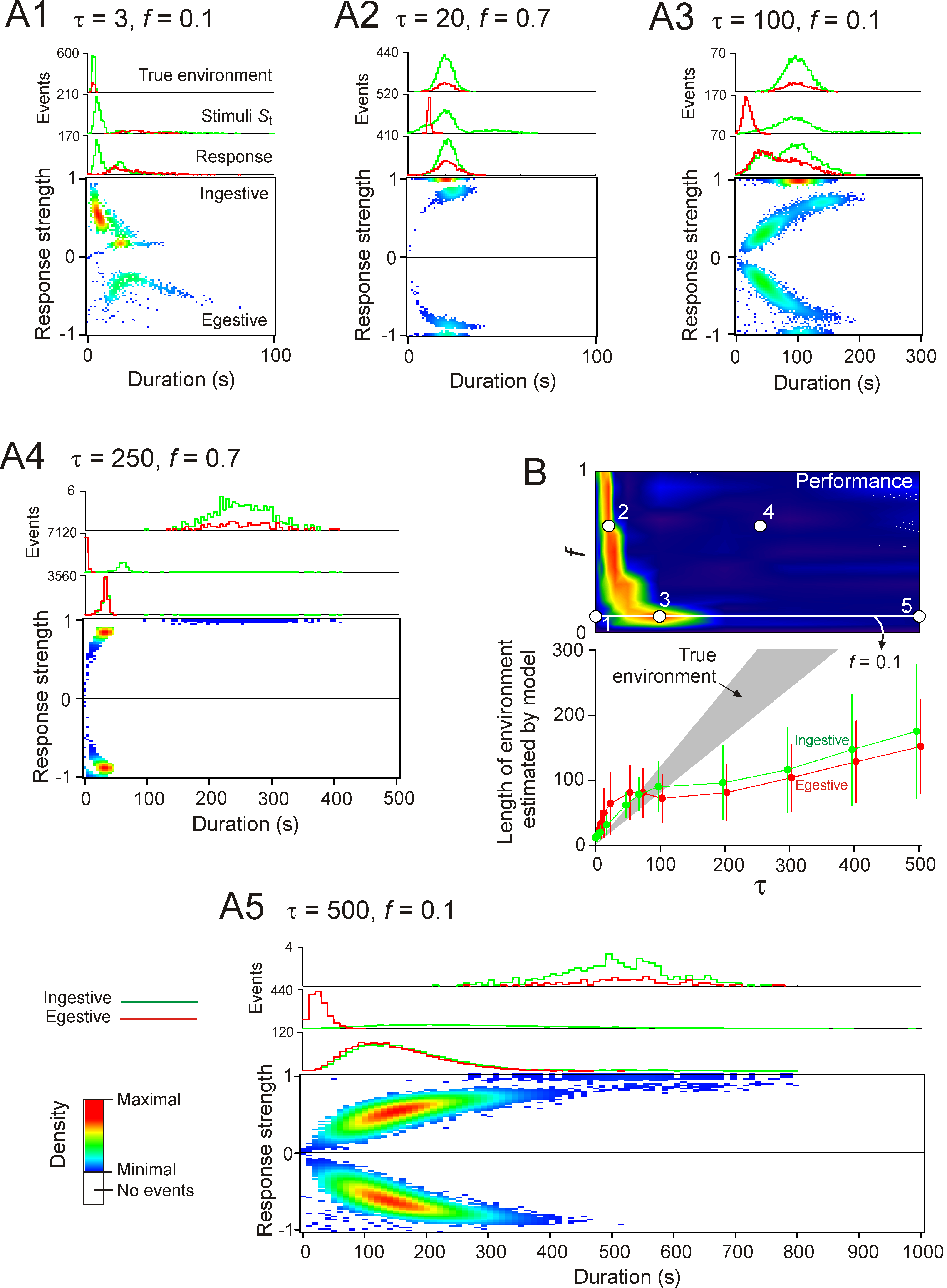

Supplement: Figure S5 — Prediction of the environment by the 2D model in Task 2. (1.71 MB TIF) [file pone.0003678.s006.tif]

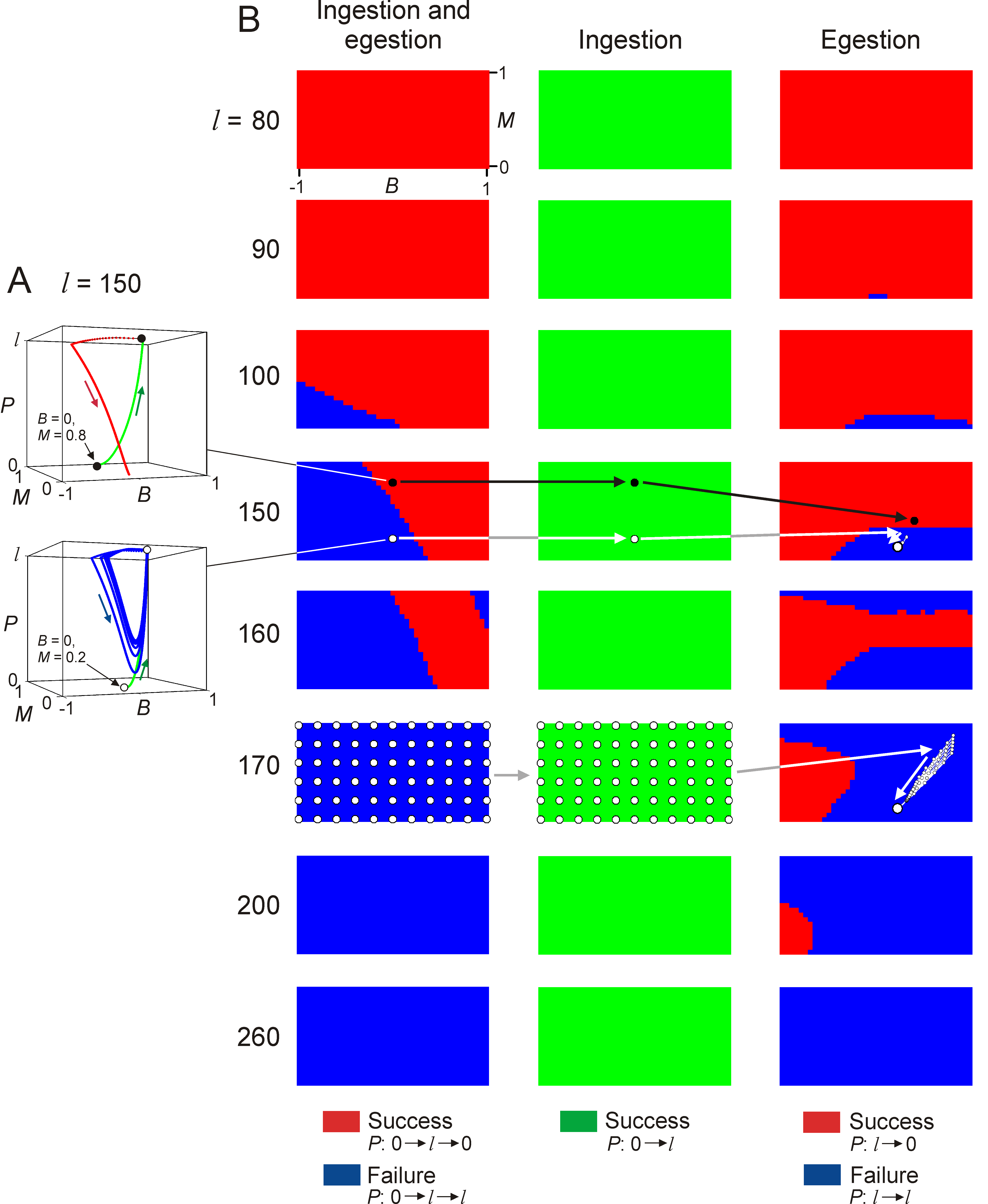

Supplement: Figure S6 — More detailed dynamical analysis of the success or failure of the ingestion and egestion of seaweed strips of different lengths. (1.39 MB TIF) [file pone.0003678.s007.tif]

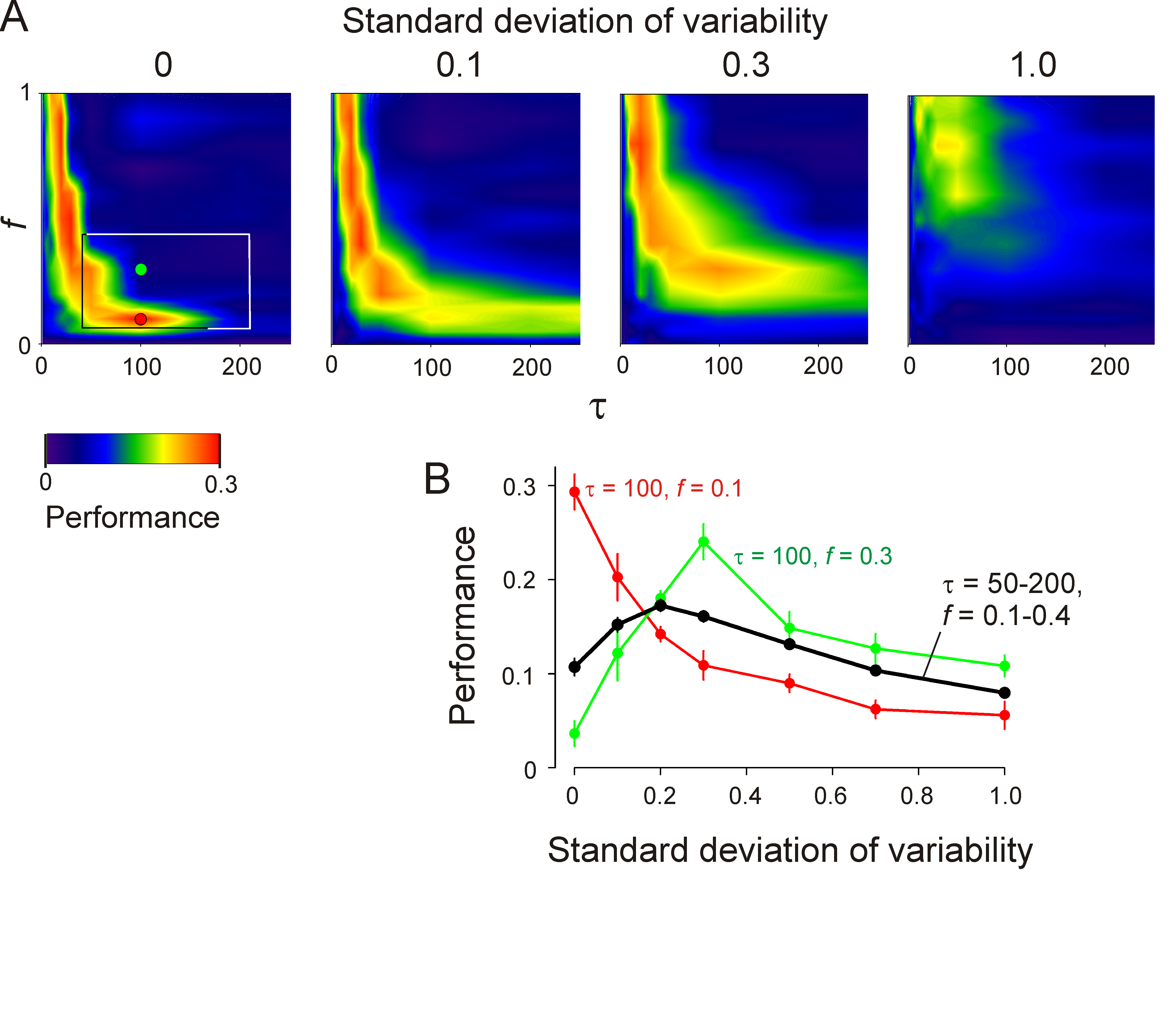

Supplement: Figure S7 — Added variability enhances the performance of the 2D model in Task 2 simulations. (1.64 MB TIF) [file pone.0003678.s008.tif]
